# Graph-based ahead monitoring of vulnerabilities in large dynamic transportation networks

**DOI:** 10.1371/journal.pone.0248764

**Published:** 2021-03-24

**Authors:** Angelo Furno, Nour-Eddin El Faouzi, Rajesh Sharma, Eugenio Zimeo

**Affiliations:** 1 LICIT UMR_T9401, University of Lyon, ENTPE, University Gustave Eiffel, Lyon, France; 2 Institute of Computer Science, University of Tartu, Tartu, Estonia; 3 Department of Engineering, University of Sannio, Benevento, Italy; Universidad Rey Juan Carlos, SPAIN

## Abstract

Betweenness Centrality (BC) has proven to be a fundamental metric in many domains to identify the components (nodes) of a system modelled as a graph that are mostly traversed by information flows thus being critical to the proper functioning of the system itself. In the transportation domain, the metric has been mainly adopted to discover topological bottlenecks of the physical infrastructure composed of roads or railways. The adoption of this metric to study the evolution of transportation networks that take into account also the dynamic conditions of traffic is in its infancy mainly due to the high computation time needed to compute BC in large dynamic graphs. This paper explores the adoption of dynamic BC, *i.e.,* BC computed on dynamic large-scale graphs, modeling road networks and the related vehicular traffic, and proposes the adoption of a fast algorithm for ahead monitoring of transportation networks by computing approximated BC values under time constraints. The experimental analysis proves that, with a bounded and tolerable approximation, the algorithm computes BC on very large dynamically weighted graphs in a significantly shorter time if compared with exact computation. Moreover, since the proposed algorithm can be tuned for an ideal trade-off between performance and accuracy, our solution paves the way to quasi real-time monitoring of highly dynamic networks providing anticipated information about possible congested or vulnerable areas. Such knowledge can be exploited by travel assistance services or intelligent traffic control systems to perform informed re-routing and therefore enhance network resilience in smart cities.

## Introduction

In the context of smart transportation, it is reasonable to consider road networks as weighted and directed graphs, to better capture road diversity (*e.g.,* length, capacity, free-flow travel time, etc.), but also as dynamic complex networks in order to model evolving traffic conditions (*e.g.,* link speed, flow, etc.) and exogenous events (*e.g.,* accidents, natural catastrophes, etc.).

The dynamic nature of transportation networks depends on multiple factors, such as travel demand, passenger behaviors, road conditions, weather-related phenomena and accidents. Similarly, recommendations deriving from trip planners might have a significant impact on traffic dynamics, by generating unbalanced traffic distribution and thus easily saturating critical areas of the network. In fact, even in the presence of smart trip planners that take into account global and real-time traffic conditions, traffic unbalance still exists due to sudden appearance of disruptions and accidents. Similarly, travel information can direct the network state towards inefficient equilibria, due the presence of unequipped users’ as well as selfish or bounded-rational behaviors from equipped ones [1, 2].

In this context, it appears beneficial to have global information about traffic conditions in order to improve the quality of local decisions by taking under control their impact on the whole transportation system. This information can be inferred from local data about traffic conditions that, today, can be easily collected via ubiquitous sensors (such as loop detectors, travel assistants, mobile phone apps, etc.) that help to monitor large urban areas. However, processing these data to provide predictive estimations of short-term traffic states and their impact on the whole network is a challenge.

Several domain-specific approaches, often leveraging simulators based on physical models of traffic propagation and people mobility, have been proposed by researchers to estimate short-term traffic conditions from monitored data [3–5]. However, these approaches struggle to scale with relevant accuracy to large urban areas, and usually do not take into account the impact of the underlying topology of the transport network and its dynamic nature [6, 7]. On the other hand, approaches based on graphs modelling represent viable alternatives to predict the behavior of dynamic transportation networks.

Current lines of research exploiting graphs in the transportation domain are mainly focused on static topological information related to nodes and intersections [8–10], so neglecting the dynamic information coming from sensors. Among the few exceptions in that sense are solutions based on graph convolution neural-network based approaches (*e.g.,* [7, 11]), which however are still at very early stages of development and suffer from extremely high computation times, thus being rather inappropriate for large-scale real-time traffic monitoring [12]. Among the metrics for complex networks that take into account both network topology and traffic dynamics, we propose dynamic Betweenness Centrality (BC), *i.e.,* BC continuously computed over a dynamic graph. In our previous work [13], we use the terms *dynamic* or *temporal* graph to denote timestamped, periodic snapshots of a network, whose topology (*i.e.,* vertex and edge sets) and edge weights may vary in time. In particular, we assume that the set of edge weights considered at time *t* are average values computed by aggregating all the different instantaneous values observed over the temporal window [*t*−*T*, *t*[, while the vertex and edge sets are those actually present at instant *t* − 1.

BC has been effectively exploited in many domains to identify the components (nodes) that are mostly traversed by information flows and therefore potential critical spots. These spots are particularly relevant in transportation networks, since they are subject to hardly predictable hazardous and cascading effects. The latter may have tremendous impacts on the operation of the urban infrastructure at large scales and with extreme rapidity.

Recent literature [13, 14] has proved that BC may identify or anticipate the appearance of critical spots in monitored transportation networks, thus justifying the application of graph-based approaches to perform ahead monitoring of traffic distribution in large-scale, dynamic road networks. An obstacle to the adoption of this approach is the computation time needed for the evaluation of BC, especially when the size of the network is large.

Several solutions have been proposed in recent years to reduce the computation time of this metric [15–17] for undirected, unweighted and static graphs, whereas approaches for highly dynamic graphs are still unsatisfactory, mainly due to the high computation time required. By *highly dynamic graphs*, we refer in this paper to graphs whose dynamic attributes, *i.e.,* edge weights, change frequently, as in the case of transportation networks.

This paper proposes an approach to compute BC values on periodic snapshots of dynamic graphs representing transportation networks. The proposed approach can be thus leveraged to promptly detect anomalies or abrupt changes of network properties and traffic dynamics, by monitoring the sudden variations of the weighted shortest paths that traverse the links of the network.

The contributions of the paper are the following:

- the adoption of dynamic BC for ahead monitoring of transportation networks conditions.
- the analysis of static and dynamic BC in different scenarios and with various graphs by exploiting a large dataset of traffic-related real observations (GPS traces of vehicles). Interestingly, and originally with respect to [18] and our other previous papers on the topic [19–22], the analysis shows the relevance of using BC for resilience enhancement and, particularly, the need for a stringent requirement in terms of computation time.
- an extensive evaluation of our algorithm in terms of performance, scalability and accuracy on large dynamic road-network graphs, showing that it outperforms other state of the art algorithms for computing BC of dynamic graphs.

Our paper, via the proposed approach, lays the foundations for a novel data-driven, complex network-based control system for supporting resilience enhancement of large-scale road networks.

The rest of the paper is organized as follows. First, we present related work. Then, we describe our model and metrics to characterize road-network vulnerability. A case study, related to the analysis of both static and dynamic BC atop a large-scale road network is presented. Finally, we conclude the work by also highlighting future directions.

## Related work

Graph models, network theory and BC, a metric originally proposed in [23], have proven to be a natural conceptual framework to study topological bottlenecks of complex systems [24, 25] from multiple domains.

Over the last decade, BC has been particularly exploited in the context of transportation networks [9, 26, 27] for traffic flow prediction [9, 10, 26, 28, 29], vulnerability detection [30–32], identification of network attacks [33] and urban activities [34], to name a few. An important aspect in transportation networks is the relative importance of paths, since the distribution of traffic largely varies depending on the nature of roads and paths [35]. Various works have therefore modeled the importance of traffic on paths by applying weights to the network edges [33, 34, 36] and have shown that centrality-based attacks can have an impact at a much larger scale on the network [24, 33] when considering weights. Wang *et al.* examine in [37] the mathematical relationships that exist between BC and edge weights in a weighted network. Specifically, the authors prove that if the network is in a weakly disordered regime, edges with smaller weights tend to carry more traffic in the network, thus introducing negative correlation between edge BC and edge weights. This is particularly interesting in the context of transportation networks that are spatial, mostly regular, graphs on which we can thus expect negative correlation between edge weights (travel times) and weighted BC.

Other works have studied the effect of high-load on roads to evaluate resilience and efficiency in transportation networks for cities in US [38], Italy [39], and China [34]. Commuters’ flow and time-schedule delays have been used for associating weights to the graph edges of the Singapore’s subway transportation network [36]. By using GPS traces, authors in [34] study two large-scale intra-city urban networks and traffic flows in the cities of San Francisco, US, and Shanghai, China.

As an important limitation, most of the existing works consider networks and their attributes (*e.g.,* weights) as static entities. This is an unrealistic modelling approach as traffic, and consequently edge weights, rapidly evolve as a consequence of change in travel demand, accidents, congestion, etc. Some approaches have been nevertheless proposed for calculating BC on dynamic graphs, such as by using hyper-graphs [40] or by a random-sampling technique [16]. In their recent work [17], Chehreghani *et al.* improve the solutions proposed in [16, 40] to efficiently update BC values on dynamic graphs. However, their approach presents several limitations. Firstly, it only addresses unweighted graphs. Secondly, the approach exhibits good performance (however in the order of seconds) on large-scale graphs only when considering mildly dynamic settings (edge/node updates are assumed to be relatively infrequent). Similarly, an efficient algorithm for incremental BC computation has been proposed in [41]. This paper proposes a framework for computing betweenness centrality in evolving unweighted graphs. The authors also claim their framework can work for directed graphs even if, for simplicity, they demonstrate their approach only on undirected network. The algorithm has good performance when BC has to be recalculated as a consequence of adding or removing only one node. Conversely, in highly dynamic settings, as those that can be expected in our application domain (*i.e.,* weighted transportation networks with multiple nodes/edges appearing or disappearing and, above all, edge weights changing every second), the approaches proposed both in [17] and [41] become largely inefficient and therefore impracticable.

BC for dynamic analysis has been proposed in [28], based on: *i)* free-flow travel time and *ii)* congested-flow travel-time. In this paper, the authors perform only an analysis of the correlation existing between BC and node congestion by distinguishing on-peak and off-peak scenarios, thus highlighting that a more dynamic definition of BC, computed over a weighted graph, can become an effective proxy for (dynamic) traffic volume information. Differently from this paper, we take a step forward by analyzing the impact of static and dynamic BC on a large graph derived from a real road network *and* by proposing the adoption of a fast algorithm to perform dynamic BC computation in short time slots with the aim of predicting possible future bottlenecks (*ahead monitoring*). In fact, we are motivated by the need of defining a monitoring system for providing travellers with dynamic information on the best paths to follow in order to globally improve network performance (*i.e.,* reducing bottlenecks and vulnerabilities) and managers with an early warning system to identify possible vulnerabilities.

The main limitation of BC when used as an indicator of vulnerability over large-scale networks is its extremely high computation time, even when computed by the fastest general solution for exact BC computation proposed by Brandes in [15] or the recent heuristics proposed by Saryuce *et al.* with their BADIOS framework in [42, 43], as proved and discussed in the performance analysis section. For instance, in the very recent work [14], BC is calculated on weighted graphs for some nodes of the network, using the Brandes algorithm to identify critical nodes with respect to traffic conditions, revealing some interesting results from the point of view of correlation with the most vulnerable nodes of the transport network. However, the proposed approach does not provide a significant advance with respect to the Brandes algorithm and no performance evaluation (both in terms of accuracy and execution time) is shown. Therefore, to perform fast computation of BC, we decided to exploit approximation [19], by excluding the computation of some shortest paths to improve performance. In [44], the authors only consider paths up to fixed length *k*. Brandes and Pich [45] also proposed an approximated algorithm for faster BC calculation by choosing only *k* ≪ *n* pivots as sources for the Single-Source Shortest Paths (SSSP) algorithm through different strategies, showing that random selection of pivots can achieve accuracy levels comparable to other heuristics. However, this approach overestimates the BC of unimportant nodes that are close to a pivot. To overcome this problem, various solutions have been proposed, *e.g.,* a generalization framework for betweenness approximation has been proposed in [46]. The idea is to scale BC values in order to reduce them for nodes close to pivots. In [47], a solution to reduce the pivots for nodes with high centrality is proposed via adaptive sampling techniques. A recent work [48] based on approximation shows large fluctuations of accuracy over the top-100 nodes on a scale-free graph. A random, shortest path based [49] approximation approach was presented in [50]. For directed and unweighted networks, an approach is presented in [51], where, similarly to [52], authors pre-compute reachable vertices for all the graph nodes.

Starting from the considerations above, we started in previous work the exploration of a different approach taking into account that the border nodes of clusters obtained through modularity-based clustering techniques are the nodes with a high value of BC since they are crossed by all the nodes inside the cluster to reach all the other nodes of the graph. Therefore, we focused on the identification of pivots, as explained later in this paper, that avoid BC errors of border nodes and the nodes outside the cluster of the pivot, for each cluster obtained by clustering the initial graph.

A first result on unweighted graphs has been presented in [53] while an improvement for reducing computation time for a static weighted graph in [18]. In [22], we have addressed the technical and technological aspects of the algorithm implementation in order to verify its scalability, when a large number of computing resources is used. This paper extends our previous work, by: *i)* performing an extensive analysis and performance evaluation of our approach on both static and dynamic weighted networks in the context of transportation; and *ii)* posing the bases for a novel proactive monitoring system that uses fast BC computation to quickly compute BC values at the current time slot and compute alternative paths for route recommendation for resilience enhancement.

## Network model and algorithm

In this section, we introduce the main assumptions we consider to model a dynamic transportation network, the related graph model, the metric we use to analyze it, and the algorithm proposed for efficient computation of this metric (BC) over large-scale, dynamic, weighted and directed networks.

### Modeling assumptions

In our study, we take into account three fundamental real-world, well-known properties of large-scale urban traffic networks: *i)* not all roads are bidirectional, especially in city centers; *ii)* traffic does not equally distribute among road segments [35, 37]; *iii)* traffic on each road segment is dynamic, as it changes according to unpredictable events and the travel demand [28, 29, 34]. Concerning our graph-based modelling, we assume that: *i)* nodes represent intersections of the road network, while directed edges correspond to directed route segments (also called links in the following); *ii)* edge weights represent, if not otherwise specified, the travel time observed to traverse the corresponding link (average over a time period or free-flow travel time depending on the analyzed scenario); *iii)* people tend to prefer the shortest (fastest) paths to reach their destinations.

The choice of travel time as edge weight stems from the importance of such variable as a proxy to identify the appearance of congestion on specific road segments. However, it is worth to remark that link travel time per se is not sufficient to identify critical network traffic condition in the near-future. In fact, road links with high travel time at a given moment might not necessarily represent critical links of the network at that specific instant or in following ones, as they might not appear on a significant number of shortest paths connecting different pairs of origins/destinations (OD). Conversely, a link characterized by a low travel time, close to free-flow conditions, could represent a critical link, as it might belong to multiple shortest routes connecting a large number of OD pairs.

The considerations above derive from the global behavior of the network: by relying on the assumption that users tend to choose shortest paths to reach their destinations, when congestion appears, or hardly predictable accidents occur on a given path, the network is not at equilibrium and people may look for alternative shortest paths to reach their destinations, possibly following the indications provided by real-time travel planners. The previous assumptions are realistic if we consider that modern vehicles are equipped with smart navigation systems and planners may significantly affect users’ route choice. This phenomenon can easily lead to grid-locks and large-scale propagation of congestion. In fact, travelers might easily saturate areas of the network (possibly still in free-flow conditions) that are central to the global functioning of the urban system (*i.e.,* nodes traversed by a significant number of alternative shortest paths and thus associated with high values of node BC).

Under the assumptions above, by continually computing node BC of large urban-scale road networks, weighted by travel times collected by sensors, control strategies could be designed to smartly re-distribute traffic flow for balancing node BC values over time with the aim of keeping the BC distribution close to the one observed in free-flow conditions. Therefore, we can conclude that: *i)* it is reasonable to analyze non-uniform distributions of (node) BC values in real-world (dynamic) road networks; *ii)* the nodes characterized by higher values of BC potentially represent critical regions (intersections) of the network, since they correspond to the nodes where traffic can be expected to most-likely concentrate in the near future; *iii)* as shortest paths depend on traffic flows, edge weights should reflect actual traffic conditions to make BC values more relevant from a dynamic system perspective; *iv)* a significant variability of centrality values has to be expected in time over the different nodes of the network due to traffic dynamics and hardly-predictable events.

By relying on the continuous quick computation of node BC over a time-varying weighted graph, such a system could be used to promptly advise vehicles about the availability of reasonable path alternatives to the shortest one, whose choice can contribute to the global improvement of network performance. Such alternatives should be computed by taking into account the current distribution of BC values in a given, possibly congested, area and by identifying the paths that allow for a more homogeneous distribution of BC values in the given area during the next time step.

### Network model

We assume the following definitions throughout the paper. Let *G*(*V*, *E*, *T*, *W*, *f*(*E*, *T*)) be a dynamic, weighted and directed graph, where *V* denotes the set of nodes and *E* ⊆ *V* × *V* the set of edges. *N* = |*V*| denotes the number of nodes in the graph. *W* represents the set of weights and *T* the set of time units. For instance, for very large networks, *T* may represent hours of the day. We highlight that the length of the considered time unit (*e.g.,* 1 hour) represents the period of observations before a new computation of BC is launched, and translates therefore into a time constraint for computing betweenness centrality. Function *f*: *E* × *T* –> *W* maps each edge *e*_*ij*_ ∈ *E* at time slot *t* ∈ *T* to a weight *w* ∈ *W*. We denote $\hat{G}$(*V*, *E*, $\hat{W}$) as a directed and weighted instance of the dynamic graph *G* related to a specific time slot $\hat{t}$ and therefore associated to a subset of weights $\hat{W}\subseteq W$. The algorithm reported in the following are related to a specific instance $\hat{G}$ of the dynamic graph *G*, *i.e.,* BC computation is iteratively performed (in a quasi-real time fashion) at the beginning of time slot $\hat{t}+1$ on the instance of the dynamic graph related to time slot $\hat{t}$.

A path *p*(*v*_*i*_, *v*_*j*_), between two nodes *v*_*i*_ and *v*_*j*_ of $\hat{G}$, consists of a set of nodes and edges that connect these two nodes. The length of a path between any two nodes *v*_*i*_ and *v*_*j*_, represented by *len*(*p*(*v*_*i*_, *v*_*j*_)), is the sum of the weights of the edges (or hops) to reach *v*_*j*_ from *v*_*i*_. If nodes *v*_*i*_ and *v*_*j*_ are directly connected, then the path length is the weight of the link, or 1 for unweighted graphs. A shortest path between any two nodes *v*_*i*_ and *v*_*j*_, denoted as *sp*(*v*_*i*_, *v*_*j*_), is the path with the minimum length, among all the paths connecting the two nodes. Multiple shortest paths may exist between the same pair of nodes, *i.e.,* multiple paths having the same length. The distance *d*(*v*_*i*_, *v*_*j*_) = *len*(*sp*(*v*_*i*_, *v*_*j*_)) is the length of the shortest path between nodes *v*_*i*_ and *v*_*j*_. We denote $\sigma_{v_{i}v_{j}}$ as the number of shortest paths between *v*_*i*_ and *v*_*j*_, while $\sigma_{v_{i}v_{j}}\left(v_{k}\right)$ represents the number of shortest paths from *v*_*i*_ to *v*_*j*_ that cross node *v*_*k*_.

Node betweenness centrality (BC) [54] is a flow metric that measures the relative number of shortest paths crossing a node.

We define betweenness centrality for weighted networks as in [33]. Let $\sigma_{v_{i}v_{j}}^{w}$ denotes the total number of weighted shortest paths from *v*_*i*_ to *v*_*j*_ and $\sigma_{v_{i}v_{j}}^{w}$(*v*_*k*_) the number of them traversing vertex *v*_*k*_, then, the weighted betweenness centrality of vertex *v*_*k*_ is defined as:

$$\begin{array}{c} BC\left(v_{k}\right)=\sum_{v_{i}\neq v_{k}\neq v_{j}\in V}\frac{\sigma_{v_{i}v_{j}}^{w}\left(v_{k}\right)}{\sigma_{v_{i}v_{j}}^{w}}, \end{array}$$ (1)

### Algorithm

We exploit modularity for clustering weighted directed graphs with the Louvain method [55, 56]. The algorithm initially searches for *small* communities to aggregate, by maximizing the modularity gain. Then, it creates a new graph whose nodes are the communities identified in the previous step. These two steps are iteratively repeated until there is no further modularity gain derived by aggregating clusters in larger communities. In our W2C-Fast-BC algorithm, the weights used to compute weighted modularity are assumed as in the notion of closeness (nodes are tighter if the interconnecting edges have lower weight, *i.e.,* distance or travel time), *i.e.,* “smaller is tighter”. This choice is motivated by the fact that we want to reduce the number of border nodes for each cluster. Therefore, we generate communities whose nodes are highly locally inter-connected with short (or fast to travel) local paths. Conversely, when computing shortest paths in SSSPs, edge weights are assumed as in the notion of length (or travel time), *i.e.,* “higher is farther”. We use a distributed variant of the Louvain algorithm for weighted and directed graphs [57, 58]: all vertices select a new community simultaneously, updating the local view of the graph after each change. Even though some choices will not maximize modularity, after multiple iterations, communities will typically converge thus producing a final result relatively close to the sequential version of the algorithm.

Given a graph $\hat{G}$, we split it into a set of clusters (*i.e.,* **C**) by using the Louvain (Alg. 1, line 2) method for weighted graphs [57, 58]. The implementation leverages a Scala parallel solution partially based on the Distributed Graph Analytics (DGA) by Sotera: https://github.com/Sotera/distributed-graph-analytics.

**Algorithm 1** W2C-Fast-BC: algorithm for fast computation of betweenness centrality (pseudo-code of the main function)

1: **function** W2C-FastBC($\hat{G},C,kFrac$)

2:  $C\leftarrow weightedLouvainClustering(\hat{G})$

3:  ${\text{bordernodes}}_{i}\leftarrow findBorderNodes\left(\hat{G},C_{i}\right)$

4:  **local*δ***_**i**_ ← *computeLocalδ*(*i*, **C**, **bordernodes**)

5:  *localBC*_*i*_ ← *localδ*_*s*_(*i*) + *localδ*_*z*_(*j*)

6:  **superClasses**_**i**_ ← *WkMeansClustering*(**C**_**i**_, **classes**_**i**_, *kFrac*)

7:  *P*_*i*_ ← *selectPivotOf*(**superClasses**_**i**_, **localBC**)

8:  ***δ***_**i**_ ← *computeδFrom*(*P*_*i*_)

9:  ***δ***_**i**_ ← (**δ**_**i**_ − **localBC**) ⋅ |**superClasses**_**i**_|

10:  *BC*_*i*_ ← *δ*_*s*_(*i*) + *δ*_*z*_(*j*)

11:  **for** *i* ← 1, |**V**| **do**

12:   *BC*_*i*_ ← *BC*_*i*_ + *localBC*_*i*_

13:  **end for**

14:  **return BC**

15: **end function**

The main result of clustering is the identification of *border nodes* (an array for each cluster). A border node is a node having at least one neighbor node in a different cluster (line 3).

Then, a parallel execution of the Brandes algorithm (based on Dijkstra) is performed inside each cluster to compute the *local BC* (lines 4-5). This computation generates the partial inner-cluster contribution to the BC of each node and additional information, such as the weighted shortest paths and the distances from a node of a cluster towards each border node of the same cluster. It is worth noting that the local contributions of BC, *i.e.,* the ones computed taking into consideration only the nodes inside a cluster as source or destination, could be affected by an error due to the so called external nodes, *i.e.,* nodes that are external to the cluster but are crossed by shortest path(s) between two nodes of the cluster. We are currently working to a solution to this problem in designing an exact version of the algorithm proposed in this paper. The preliminary results are reported in [59].

The information above is used also to identify the nodes inside each cluster *C*_*i*_ that equally contribute to the dependency score of each node of the graph (class of equivalence, see [20, 53] for more details). In particular, a class of equivalence inside a cluster *C*_*i*_ is defined with reference to the values of dependency score (BC contribution) computed on the nodes outside *C*_*i*_ with an SSSP exploration started from a node of the class: all nodes in the class equally contribute to the BC of the nodes outside *C*_*i*_. Taking into account that nodes belonging to the same class produce the same dependency score on each node of the graph outside cluster *C*_*i*_, one representative node should be identified as a source node (called class *pivot*, line 7) for applying Dijkstra’s algorithm (line 8) to perform SSSP on nodes ∈*G* − *C*_*i*_ in order to compute the contribution of all the nodes of the class by multiplying the dependency score due to the pivot by the cardinality of the pivot’s class.

The partial dependency score calculated for the pivot is then multiplied by the cardinality of the pivot class (line 9). This method avoids re-applying Dijkstra’ algorithm to another node of the same class, thus ensuring fast calculation of BC if *P* ≪ *N*, where *P* represents the set of pivots selected and *N* represents the number of nodes of the graph. However, since we multiply by the cardinality of a class the dependency scores of the nodes both outside and inside *C*_*i*_, we introduce an approximation error since the simplification applies only to the nodes outside *C*_*i*_. We are working also to remove this source of error in the exact version of the algorithm [59].

To further reduce the computation time, we have extended the concept of *class* by introducing *super classes* through an additional clustering operation inside each initial Louvain-derived cluster (line 6). A super class is a group of classes belonging to the same Louvain cluster; it is obtained via a second clustering operation (K-means) applied to the nodes of each Louvain cluster, by considering as the nodes’ features: *i)* the normalized distances from the Louvain cluster’s border nodes; and *ii)* the amount of shortest paths towards them. This grouping is aimed at reducing the amount of classes (and consequently the number of pivots) but introduces further approximation since the nodes belonging to a superclass could contribute with different scores to the BC values of the nodes of the graph.

To perform class grouping, we exploit a parallel implementation of the *K*-means algorithm by using a different *K* for each initial Louvain cluster. *K* is defined as a fraction (*K*-*Fraction*) of the initial number of classes belonging to each Louvain cluster. For example, by considering a *K*-*fraction* equals to 0.4, the algorithm adopts a 0.4 fraction of the number of classes in each Louvain cluster. This way, we are able to drive the behavior of the algorithm towards the desired computation time. However, when the computation time decreases, the approximation worsens, as deeply illustrated in our previous work [19, 20, 53].

Differently from the first two sources of errors, this last one can not be removed since it is induced to relax the constraints of class identification to have larger classes and consequently a lower number of pivots. However, it is important to highlight that removing errors has an impact on performance; this consideration leads to the conclusion that approximation and computation time should be considered as a whole and consequently when approximated results are acceptable, as in the domain we are discussing in this paper, the current version of W2C-Fast-BC is to prefer. The final value of BC is obtained for each node by summing up all partial contributions (produced by the reduce operation of line 10) with local BC values (lines 12).

## Case study: Traffic on a road network

In this section, we discuss how dynamic betweenness centrality can help in understanding transportation traffic dynamics and providing insights for predicting traffic flows.

### Dataset

**Fig 1:**
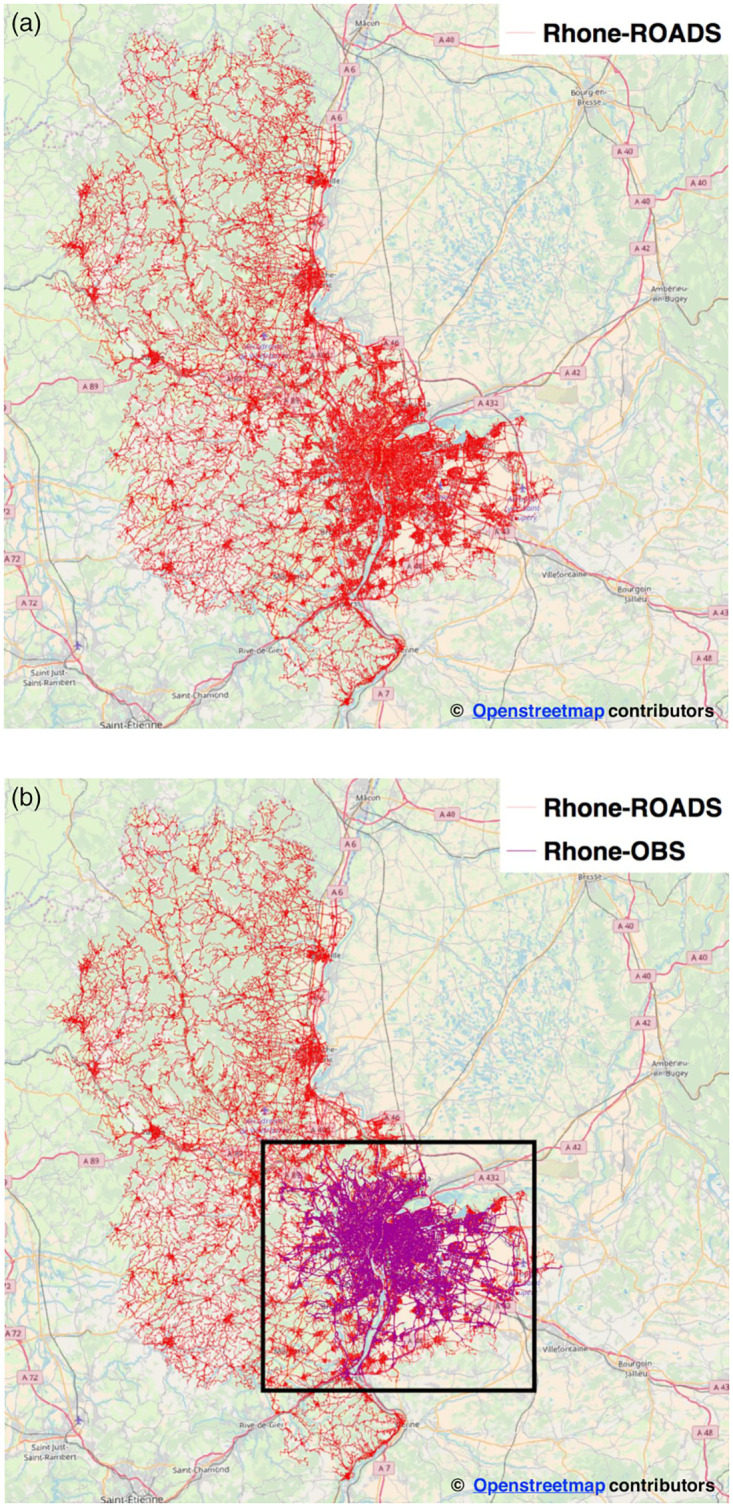
The Rhone-department road network. Maps throughout this research article were created using open-source data from OpenStreetMap and OpenStreetMap Foundation, which is made available under the Open Database Licence. Map tiles are from OpenStreetMap cartography, which is licensed as CC BY-SA (see https://www.openstreetmap.org/copyright). (a) Rhone-ROADS: the Rhone-department network, graphically shown as an undirected and unweighted graph (b) Rhone-OBS: The Rhone-department sub-network shown on top of the Rhone-ROADS. The graph only includes edges with at least one observed elementary taxi trip (in purple).

For our analysis, we consider a very-large directed graph, namely **Rhone-ROADS**, corresponding to the entire road network of the Rhone department, France. The graph includes the whole agglomeration of Lyon and its surroundings, with a geographical extent of approximately 3,300 *Km*^2^. This dataset was created using digital maps supplied by the French National Institute of Geographic Information (IGN). The network consists of 117,605 nodes and 248,337 edges. By extracting the largest connected component of the resulting network, the final undirected and unweighted graph (see Fig 1a) included 75,474 nodes and 96,406 edges (see Table 1 for the main characteristics of the datasets).

**Table 1:** Description of the considered datasets.

Name	Description	Size	Dataset Main Attributes	Source
**Rhone-ROADS**	Multi-attribute, directed, static graph of the Rhone department road network.	117,605 nodes and 248,337 edges.	Edge attributes: road segment length, width, number of lanes, speed limit, importance of the road segment (from 1 = max to 5 = min), post-code of the road segment area (INSEE), link geometry.	IGN
**Rhone-TAXIS**	Map-matched, time-stamped, geo-referenced trips of floating taxis on working days of March, April and May 2011.	5,662,844 GPS geo-referenced elementary trips related to 103,639 unique taxi trips.	Elementary-trip attributes: unique taxi’s trip identifier, GPS-logged coordinates of the segment starting point, GPS-logged coordinates of the segment arrival point, segment travel time.	Radio Taxi
**Rhone-OBS**	Multi-attribute, directed sub-graph derived by joining the Rhone-TAXIS dataset and the Rhone-ROADS network: each edge of the subnetwork has at least one elementary trip associated to it.	35,940 nodes and 59,132 edges.	Edge attributes: static attributes from Rhone-ROADS; per-edge median speeds derived from the elementary trips (24 median speed values for each edge, corresponding to the median of all speeds observed over the edge during the twenty-four 1h-time-slots from 00:00 to 23:00), as derived from Rhone-TAXIS.	Derived dataset

By considering the graph only as undirected and unweighted, many relevant properties of the road network and its dynamics could be missed. Therefore, in the first part of our evaluation, we consider multiple weighted, directed and static graphs that have been derived from the Rhone-ROADS network by selecting some of the available topological attributes (*e.g.,* road segment lengths, capacity, free-flow travel times, etc.) as weight for the edges. Moreover, to evaluate our algorithm for efficient BC computation also in more realistic dynamic settings, we leverage an additional dataset for extracting reliable time-varying traffic information for a portion of the Rhone-ROADS network.

Our second dataset, namely **Rhone-TAXIS**, contains anonymized GPS traces of taxi trips, observed over the Rhone department. The source dataset has been collected by the French operator Radio Taxi via a fleet of approximately 400 taxis, during 2011-2012. The dataset logs geo-referenced taxi trips, segmented according to a variable sampling interval (between 10 and 60 seconds), with a global average of 800,000 measurements per day. We deem *elementary taxi trip* each measured trip segment from the same taxi trace. An elementary taxi trip includes the time-stamped start and arrival GPS positions of the associated taxi. These measures permit to estimate the travelled distance, and therefore the instant speed, on the traversed road segment via a map-matching operation. Map-matching has been performed via the open-source Mapillary Python map-matching library https://github.com/mapillary/map_matching. As an indicator of traffic dynamics, we adopt in the following the median speed observed over each road segment (*i.e.,* an edge of the network) during a fixed-duration of observation (*e.g.,* 1-hour time slots). In order to improve the quality of the Rhone-TAXIS dataset and properly compute our traffic indicator, we have filtered out elementary trips with unrealistic speeds (*i.e.,* higher than 130 Km/h).

A preliminary analysis of our dataset has shown a relatively low number of observations especially during evening and night-time (*i.e.,* the daily average of elementary trips per-hour is approximately equal to 8748.5, with very high spatio-temporal variance and several time slots with 0 observations during the period March-May 2011). Therefore, to build realistic traffic dynamics, we perform an aggregation along the temporal dimension by reconstructing a *typical working day* from the whole set of elementary trips. Thus, we extract hourly median speeds for all edges with observations and generate a 24-sized array of median-speeds (*i.e.,* one per 1-hour time slot of the typical day) for some of the Rhone-ROADS network edges. We choose the median operator as it is traditionally more robust than the average one and therefore more appropriate to derive realistic traffic measures. Clearly, the number of edges with median speed changes according to the spatio-temporal distribution of elementary trips on the Rhone-ROADS graph.

**Fig 2:**
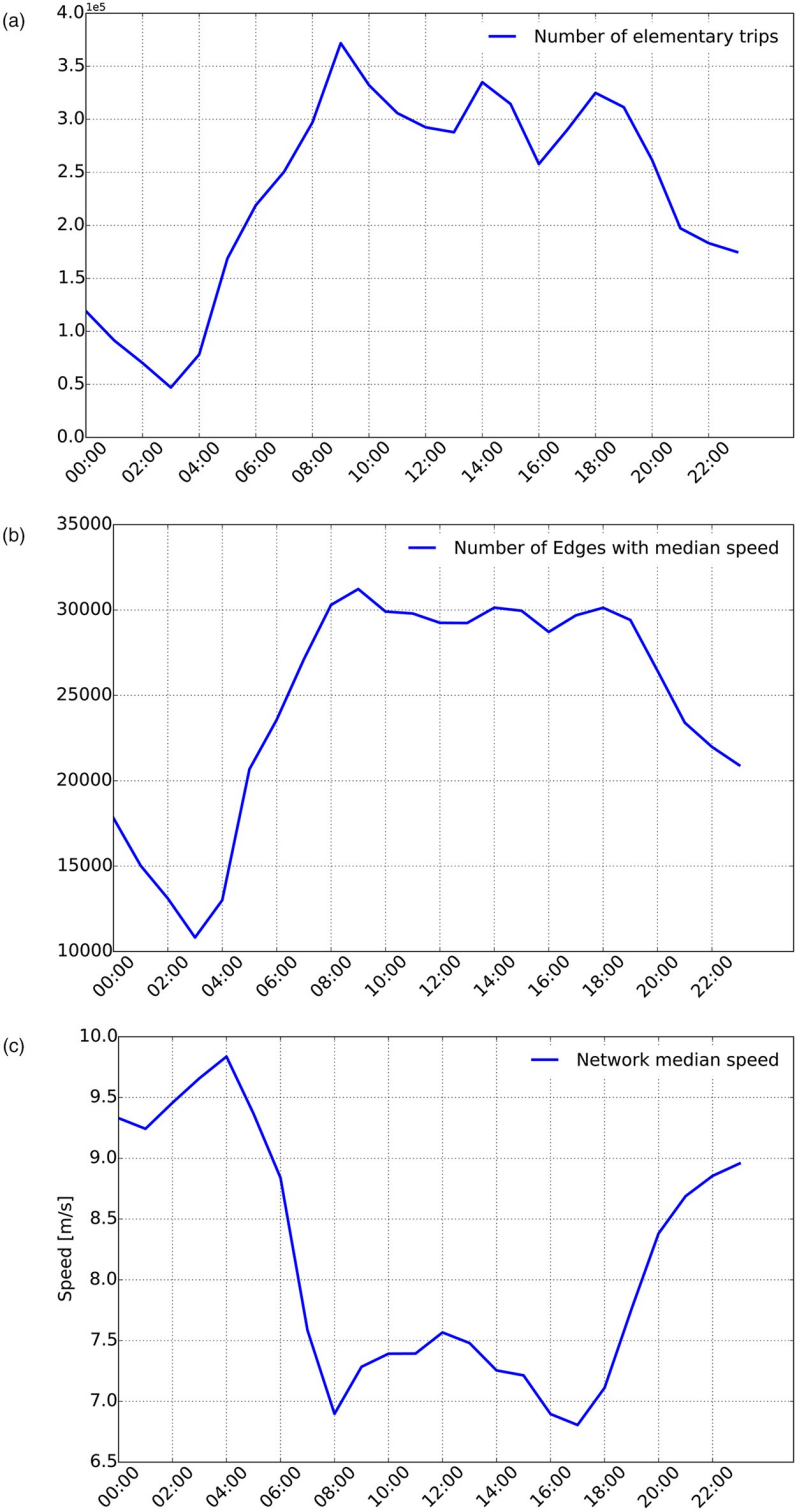
Spatio-temporal characterization of taxi observations. (a) Hourly number of elementary trips for the typical working day (b) Number of edges from the Rhone-ROADS with median speed (c) Evolution of the network median edge-speed over time.

In Fig 2a, we report the evolution, over each hour of the typical working day, of the number of observed elementary trips in the whole Rhone-ROADS network. Fig 2b describes instead the variation in time of the number of edges for which it was possible to compute the median speed. Finally, Fig 2c shows the temporal profile of the median speed, aggregated over the whole network in each time slot (*i.e.,* median of all the median speeds associated to the edges at a given hour). The network median speed profile appears to be realistic from a traffic perspective, with the highest spikes at night/early-morning time (*e.g.,* around 4:00) and the lowest ones at morning and afternoon rush hours (*e.g.,* 8:00 and 17:00). We underline the fact that this aggregation step only stems from the limited size of the taxi fleet associated to our dataset (approx. 400 vehicles) and the consequent low spatio-temporal resolution of the observations. A larger dataset would have allowed us to refine the analysis by increasing both the temporal (*e.g.,* 10-minutes slots) and the spatial (*e.g.,* a larger portion of the Rhone-ROADS graph) resolution.

Regarding the spatial dimension, Fig 1b graphically presents the sub-network (*i.e.,* the purple framed area) of the Rhone-ROADS graph for which at least one elementary trip is available during the typical working day. We call this sub-network **Rhone-OBS** graph, as reported in Table 1. Rhone-OBS comprises 35,940 nodes and 59,132 edges. With respect to Fig 2b, it is worth noting that the maximum number of edges with median speed is observed at 9:00 and corresponds to approximately 31,000 edges. The 59,132 edges of the considered Rhone-OBS graph derive from the fact that, during the 24 hours, the set of edges with median speed may vary significantly. Thus, the 59,132 edges represent the total number of different edges with at least one non-null value of median speed, over the 24 hours.

From visual inspection of Fig 1b, it appears that most of the observed elementary trips are condensed within the city of Lyon, as the activity of the taxi operator. Taxi usages from customers, appear to be concentrated around the urban area of the city and its airport area in the South-Eastern region. In addition, a few observations are available in the outskirts and within rural areas. While the Rhone-OBS graph is used in the following two sub-sections (*BC on Static Graphs* and *BC on Dynamic Graphs*) to analyze the significance of computing BC on weighted and dynamic graphs, it is worth to highlight that such graph is too small to evaluate the performance of our approach in a realistic large-scale urban road network scenario, which is expected to justify the adoption of our W2C-Fast-BC algorithm for ahead road network monitoring. Therefore, in order to prove the efficiency of our solutions with large scale networks, we leverage a dynamic graph having the same size of the Rhone-ROADS network in the *Performance Analysis* section. In particular, for those edges without GPS observations, traffic dynamics are derived from the smaller Rhone-OBS graph via a spatial interpolation technique discussed in the *Dynamic Graphs* sub-section of the *Performance Analysis* section.

### BC on static graphs

As a preliminary step for the evaluation of BC on *dynamic* graphs, we analyze the Rhone-OBS graph in three *static* configurations, i.e.: *i)* undirected and unweighted, *ii)* weighted according to the length of each road segment and directed according to road direction, *iii)* weighted according to Free-Flow Travel Time (FFTT) and directed according to road direction. The objective of such an analysis is to shed light on the usefulness of the BC metric with weighted (and directed) graphs, thus proving that different kinds of weights may grasp different notions of vulnerability. A similar analysis is conducted also in dynamic settings, as reported in the next sub-section. For consistency, both the static and the dynamic analyses have been performed on the same Rhone-OBS graph.

**Fig 3:**
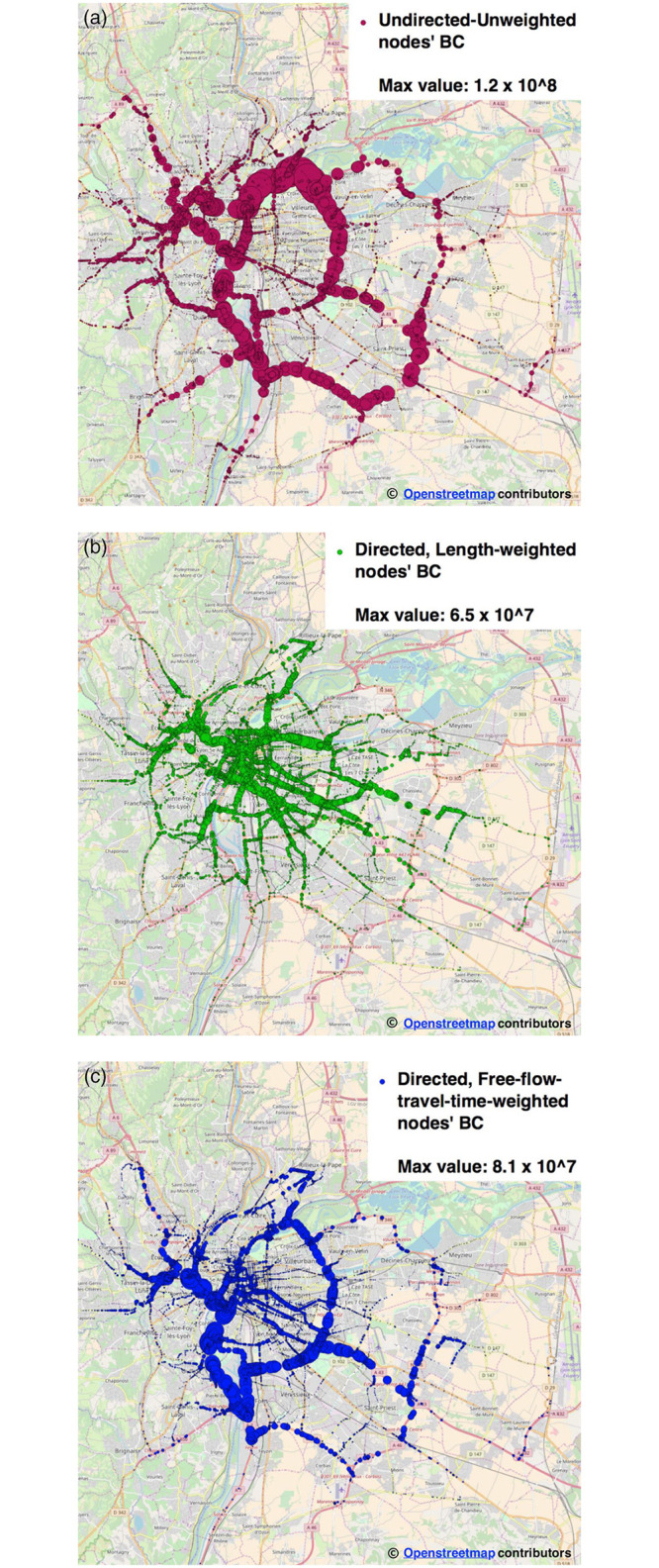
Three static topological instances of the Rhone-OBS graph: Comparison of BC values. (a) Undirected, unweighted (circle size proportional to node’s BC) (b) Directed, length-weighted (circle size proportional to node’s BC) (c) Directed, free-flow-travel-time-weighted (circle size proportional to node’s BC).

The three static configurations of the graph are graphically represented in Fig 3: for each node, we report BC values as circles with diameter proportional to the value of BC. Edges are filtered out for the sake of readability.

The visual inspection of the different figures of BC, makes it evident the effect of using different weights for BC computation. Particularly, it can be noticed that on the undirected, unweighted version of the Rhone-OBS graph (Fig 3a), top-BC nodes are mostly positioned over the city ring road and on top of major highways; urban arterials host the majority of top-BC nodes in the case of the road-length weighted directed graph (Fig 3b); finally, for the directed, FFTT-weighted instance of the Rhone-OBS graph (Fig 3c), top-BC nodes can be observed on both arterials and ring roads, with a more homogeneous distribution of BC values with respect to the unweighted, undirected case.

### BC on dynamic graphs

**Fig 4:**
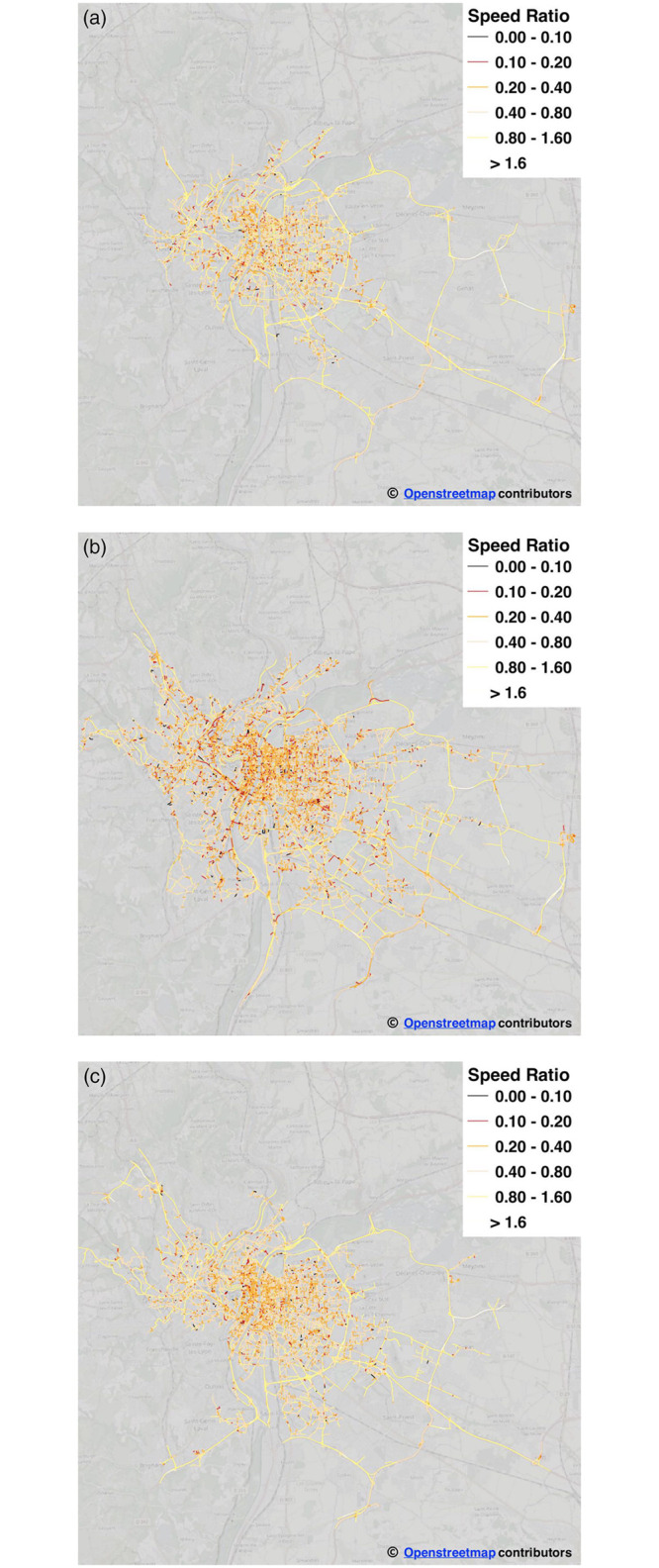
The dynamic taxi graph: Median-speed-to-max-speed ratio at different hours of the day. Edge color (from black/red to yellow) indicates higher speed-ratio, *i.e.,* reduced congestion) (a) 05:00 (b) 08:00 (c) 20:00.

In order to produce a dynamic graph, the Rhone-OBS graph has been leveraged to extract multiple weighted graph instances, depending on the specific time slot we consider in our analysis. Several instances of the Rhone-OBS graph, related to different hours of the typical day, are graphically shown in Fig 4.

To extract the graph associated to a specific time slot *t* (*e.g.,* *t* = 05:00 in Fig 4a, *t* = 08:00 in Fig 4b, etc.), we retain only the edges with non-null value of the median speed at time *t*, as estimated from the taxi trips related to the same time slot *t*. The final graph associated to time slot *t* includes the median speed observed at *t* over each retained edge. Thus, we calculate the weight of each edge as the estimated travel time to cross the corresponding road segment, by dividing its length by the estimated median speed. This produces the final weighted graph used for computing the weighted shortest paths. We remark that this approach is conceptually equivalent to an on-line operational situation, where the graph naturally emerges from sensor-collected data used to continuously compute up-to-date traffic information on each edge with given periodicity.

In Fig 4, we also graphically reports the traffic dynamics associated to the different snapshots of the observed road network. Edges are colored as a function of the *speed ratio*, defined as the ratio of the observed median speed at time *t* and the maximum speed (*i.e.,* speed limit) allowed on the edge. Black and red colors indicate highly-congested situations on the edge, *i.e.,* lower values of the speed ratio, while greens and blues indicate a smooth, non-congested situation at time *t*. The figures clearly highlight that congestion is less intense during early-morning and late-evening time slots. However, as also previously reported in Fig 2a, a lower number of taxi trips is observed during these moments of the day. Moreover, extremely low observations take place during deep-night and very-early morning time (i.e.., from 01:00 to 05:00) as a consequence of low taxi usages, which also reduces the statistical relevance of the estimated speeds. Fig 2a and 2b indicate that the morning peak hour period (6:00—11:00) contains the largest number of observations (and therefore the largest observed traffic flow) from our dataset, and that such observations are mostly spread over the network, with around 200,000 to 380,000 individual hourly trips, concerning approximately 25,000 to 31,000 edges of the network. Similarly, regarding flow dynamics, Fig 2 confirms that during this time interval, traffic flow is highly sensitive to congestion, following the classic pattern associated to peak hours: the highest median speed of approximately 9 m/s, observed at 6am, drops down to a local minimum of 6.9 m/s at 8am and gradually comes back to a local maximum of 7.4 m/s at 11am, due to reduced traffic flow and congestion reduction. Therefore, we focus the rest of the evaluation on the more statistically relevant graph instances related to time range 06:00 − 11:00 which also represent the ones most sensitive to traffic flow dynamics. The repetitive nature of traffic congestion further justifies the hypothesis of focusing on only one peak hour of the day.

**Fig 5:**
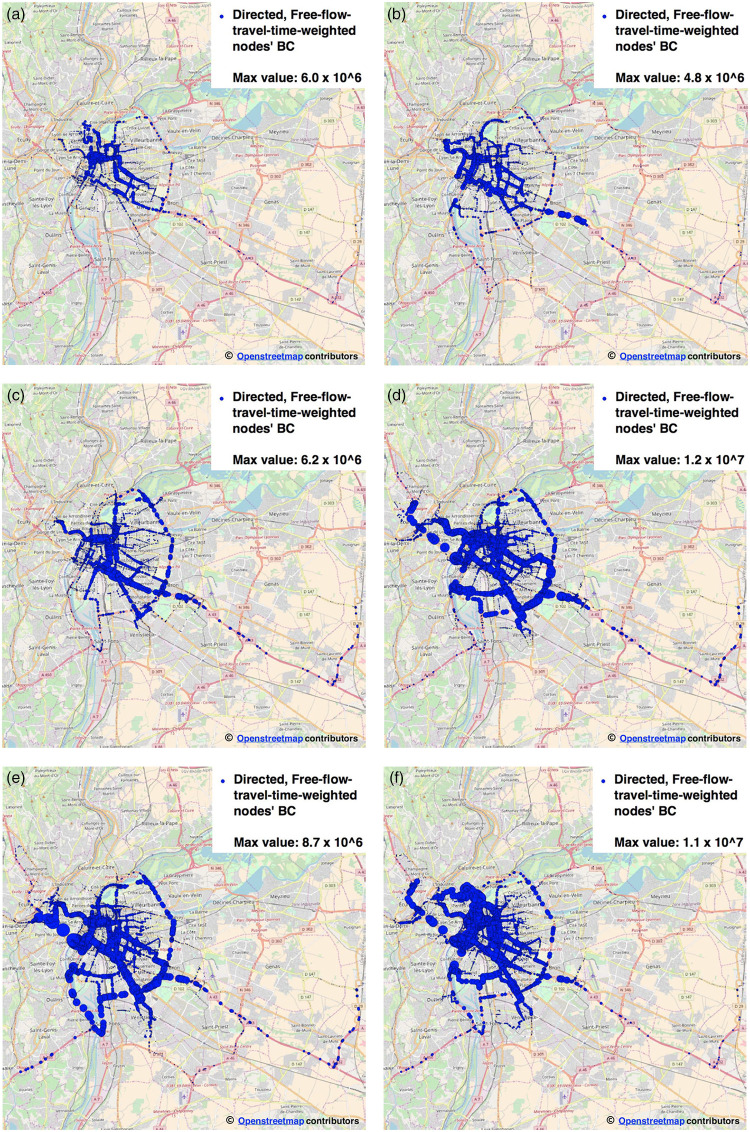
Nodes’ travel-time-weighted BC over the dynamic graph in the time range [05:00–10:00]. The size of each circle in the subplots is proportional to node’s BC. (a) 06:00 (b) 07:00 (c) 08:00 (d) 09:00 (e) 10:00 (f) 11:00.

**Fig 6:**
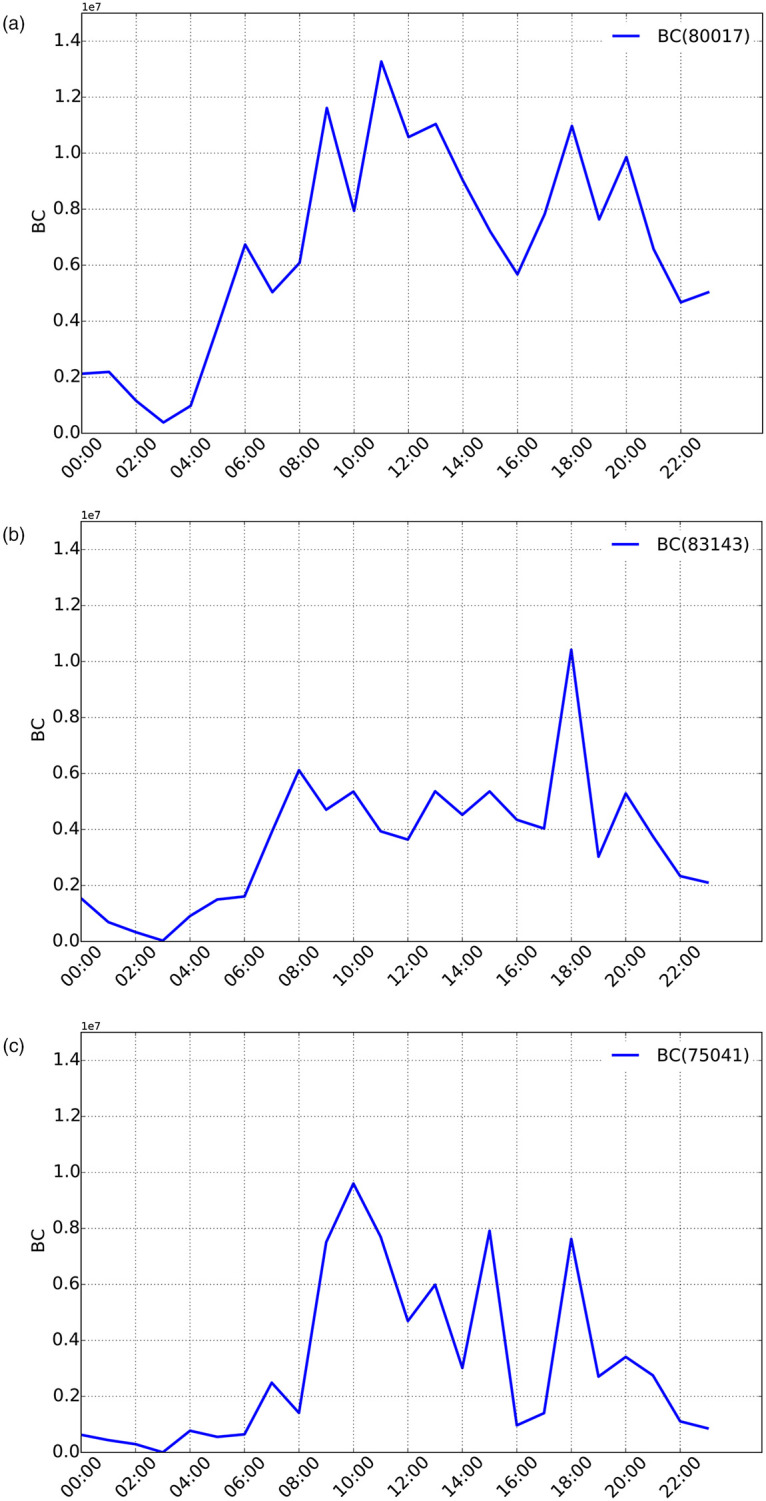
Evolution over time of the top-BC node for some time slots of the dynamic taxi graph. (a) Node with the highest BC at 06:00: evolution of its BC over time (b) Node with the highest BC at 08:00: evolution of its BC value over time (c) Node with the highest BC at 10:00: evolution of its BC value over time.

In Fig 5, we present the spatial distribution at different hours of high-BC nodes, computed on the weighted dynamic graph. The visual inspection of the sub-figures denotes high variability in time and space of the nodes with higher values of travel-time weighted BC (termed TTBC in the following), as also confirmed in Fig 6, which depicts the evolution over time of TTBC value associated to the node with the largest value of TTBC at given time slot.

These results provide first hints on the importance of using dynamic, weighted graphs in the computation of BC as well as the need for a rapid algorithm for computation of up-to-date BC values. In that sense, the figures unfold interesting dynamics of people’s mobility in the city of Lyon: during morning peak hours (Fig 5c and 5d), higher TTBC nodes concentrate along the high-speed Lyon ring road, which thus represents one of the most important (and therefore critical) connecting roads of the city, being traversed (in those time slots) by the largest number of the weighted shortest paths. Similarly, some specific arterials traversing the city center also appear as associated with higher TTBC. It is worth noting that such critical roads change frequently, depending on the specific time slot, thus unveiling high traffic dynamics at morning peak hours.

**Fig 7:**
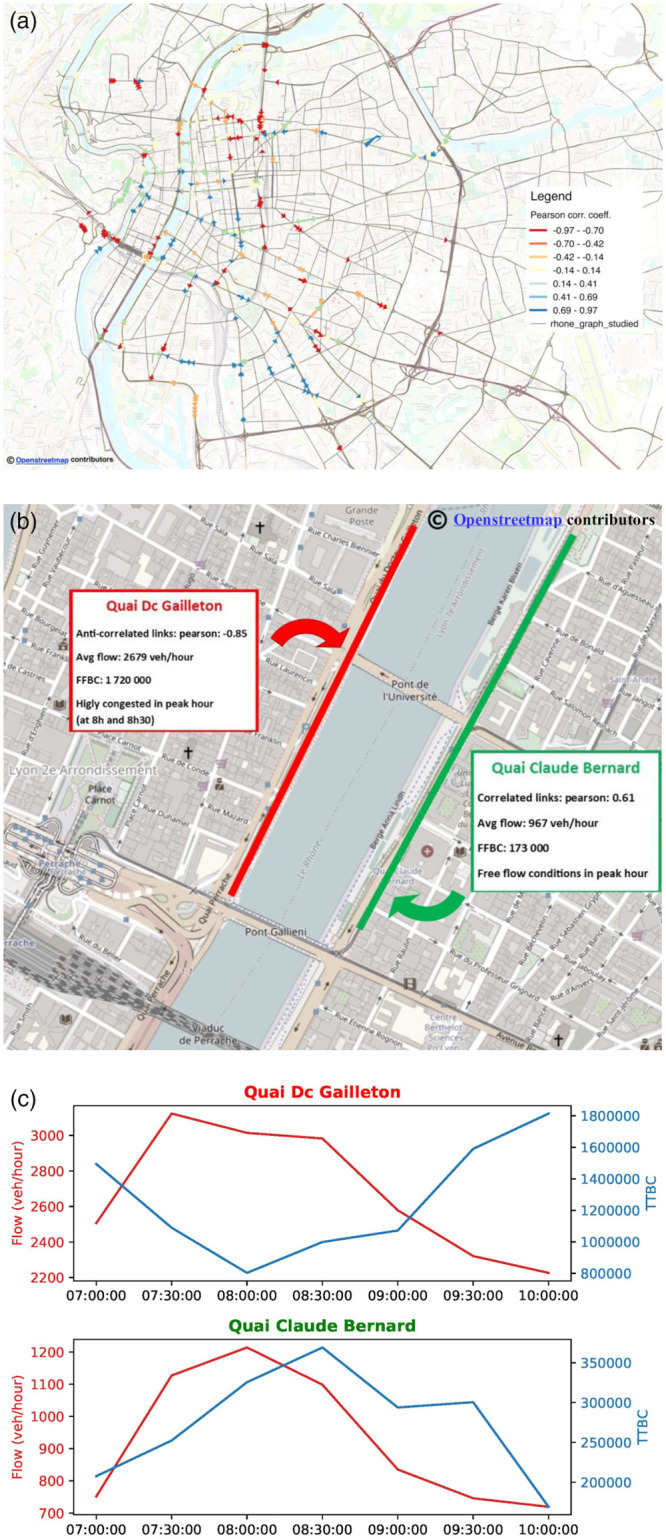
Per-edge temporal correlation between BC and flow (a) and zoom on a specific region (b,c). (a) Per-edge temporal correlation (only edges equipped with loop detectors have a non-null value) (b) Zoom on an area with two roads: Qaui Dr. Gailleton and Quai Claude Bernard (c) Evolution of the TTBC and flow on the road: Qaui Dr. Gailleton and Quai Claude Bernard.

In order to dig deeper into the interactions between TTBC and traffics dynamics, we performed a more specific analysis of the *temporal correlation* between dynamic BC (TTCB) and traffic flows. The latter are derived from an additional dataset provided by the Lyon municipality. Flow data are collected via vehicle counting sensors (*loop detectors*) installed along multiple road segments of the city. Such sensors reports the hourly flow values observed along a subset of the edges of our Rhone-OBS graph. Hence, we calculate the linear correlation score (which can be either positive or negative) for each edge *l* of the network. This correlation is computed by considering, for a given edge *l* of the graph, the vector corresponding to the per-edge TTBC values over the considered time slots and the vector of the corresponding observed vehicle flows (measured as veh/hour). Due to the sparsity of the available flow data (loop detectors are only available for a limited number of edges of our Rhone-OBS network), the reported correlation analysis refers to edge TTBC and not to node TTBC, which is the metric computed by our W2C-Fast-BC algorithm. However, the main insights from such correlation analysis can be safely generalized to node’s TTBC as well [28, 34]. In fact, it is possible to compute per-node aggregate traffic flows by summing up the flow values available on incoming/outgoing edges and observe similar behaviors as those reported in Fig 7c. A more detailed correlation analysis is outside the scope of this paper and will be matter of future work. The spatial map reported in Fig 7a highlights the heterogeneity of this correlation.

We analyze the detail of these dynamics (related to dynamic BC and flows) by focusing on a specific region of the analyzed graph including two roads with a mirror behavior in terms of temporal correlation, *i.e.,* *Quai Dr. Gailleton* (QDG) and *Quai Claude Bernard (QCB)*. QDG is a road segment typically attracting larger flow than QCB, as confirmed by an observed average flow of QDG equals to 2,679 veh/hour, significantly higher than the one observed on QCB (967 veh/hour). From the available speed data, we know that congestion is typically observed on QDG at 2 different time slots, *i.e.,* 08:00 and 08:30. Therefore, an increase of travel time is also observed on the link at these time slots. This is coupled to a decrease of TTBC, which exhibits therefore an anti-correlation behavior. Conversely, the QCB link, which is characterized by a lower demand, appears to be in free-flow conditions during the whole observed time period. As travel time is higher for QDG in congestion period, QCB becomes therefore a viable, attractive alternative for drivers to avoid congestion on QDG. In line with expectations, we observe indeed a TTBC increase, as well as an increase of flow on such road segment. This explains why the QCB edge exhibits a highly positive temporal correlation during the analyzed time slots (08:00 and 08:30). After the congestion phase (08:00 and 08:30), the flow globally decreases in the area. As QDG becomes more and more fluid, the corresponding travel time decreases to free-flow travel time and TTBC increases, *i.e.,* thus again maintaining an anti-correlated tendency. Contextually, QCB appears to lose its attractiveness compared to QDG as the flow decreases on this edge. TTBC decreases as well confirming the positively correlated trend. The analysis is confirmed by the evolution of the flow and the TTBC, which are known from the available data for both QDG and QCB as reported in Fig 7c.

To summarize, BC computed on static weighted graphs (*e.g.,* free-flow travel time weighted) can provide information on critical edges, *i.e.,* road segments on which a high flow and possible congestion should be expected. More interestingly, by studying BC on a dynamic graph (*i.e.,* travel-time weighted as in TTBC), it becomes possible to spot different kind of behaviors. Dynamic BC appears to be highly anti-correlated with respect to traffic flow dynamics in areas that are critical by nature (*e.g.,* high-capacity attractive roads) where, even if congestion can be occasionally observed, relevant flows will still be observed, while travel time may increase thus reducing TTBC. On the other hand, dynamic BC will be highly positively-correlated to traffic flow in buffer areas that become more attractive in term of travel time only when nearby roads become congested, thus collecting higher flows. This observation leads to the possibility of understanding how traffic flows will distribute in the near future based on BC values computed on previously collected travel times, which allow to weigh the graph. Therefore, the result of BC computation and its interpretation represent a sort of anticipated monitoring of traffic flows distribution.

Finally, an intermediate situation exists, with “neutral” areas (mildly positively or negatively correlated in terms of TTBC and flow) being characterized by either low or medium/high traffic demand and usually capable to dispatch such flow, without becoming congested. It is worth to remark that the considerations above further confirm the need for providing an efficient solution to rapidly compute BC on large-scale and very dynamic weighted graphs.

### Performance analysis

We exploited our W2C-Fast-BC algorithm [18] to perform static and dynamic analysis of the metropolitan road network of Lyon. W2C-Fast-BC is written in *Scala* with the *Apache-Spark* framework, leveraging multi-core processing for parallel execution. In particular, since our algorithm computes approximated values of BC, we take the error under control by calculating it for each value of the K-fraction parameter of the algorithm. It is worth noting that we are interested in identifying the top-n critical nodes of a road network and in the following we consider *n* = 1000, which means that we want to discover with a global analysis the first 1000 critical intersections of the road network. Consequently, the average error that we compute is related to only the first 1000 nodes of the analyzed graph.

As a preliminary step to evaluate the performance of W2C-Fast-BC, we have compared our approach to both Brandes [15] and BADIOS [42, 43], which represent the two most relevant approaches for fast computation of exact betweenness centrality from the state-of-the-art. To perform a fair comparison and remove any bias due to the adopted programming language and the associated runtime environment, we have compared our W2C-Fast-BC implementation to a custom Scala implementation of Brandes. Concerning BADIOS, we have considered the open-source C++ implementation provided by its authors and compared it to the C++ implementation of Brandes, included within the BADIOS framework for benchmark purposes.

The reported analysis has been performed in sequential mode for two reasons: first of all, a sequential execution permits to clearly quantify the benefit of our technique with respect to Brandes only as a consequence of the reduced number of SSSP explorations, whose number corresponds to the cardinality of the identified set of pivot nodes. Secondly, the available implementation of BADIOS does not support parallelism. As another important limitation, the available implementation of BADIOS does not implement any support for weighted graphs. Thus, we have considered an unweighted version of the Rhone-ROADS graph from Table 1 for this performance benchmark. We highlight that BADIOS has been setup in our tests to exploit all of the different optimisation techniques proposed by the authors.

Table 2 reports the speedup values (computed as the ratio of the sequential execution time of the analyzed solution to the sequential execution time of the considered Brandes implementation) obtained when comparing W2C-Fast-BC and BADIOS with respect to the corresponding implementation of Brandes (*i.e.,* the Scala and C++ implementation of Brandes, respectively). First of all, it is worth to note that our W2C-Fast-BC solution largely outperforms Brandes with a speedup larger than one in all considered configurations. In particular, we highlight the higher speedups that can be achieved when selecting a K-fraction parameter lower than 0.5. Regarding BADIOS, a speedup of approximately three is observed on the Rhone-

**Table 2:** Performance evaluation in sequential settings: Comparison of W2C-Fast-BC with respect to Brandes [15] and of BADIOS [42, 43] to Brandes.

W2C-Fast-BC configurations		W2C-Fast-BC avg. perc. error (top- 1000 nodes) [%]	Speedup of W2C-Fast-BC to (Scala) Brandes	Speedup of BADIOS to (C++) Brandes
K	# pivots			
0.01	972	0.93	**29.79**	3.00
0.1	9,684	0.175	**8.22**	
0.2	19,368	0.089	**4.54**	
0.3	29,047	0.050	**3.35**	
0.4	38,732	0.043	2.45	
0.5	48,445	0.038	1.96	
0.6	57,905	0.023	1.70	
0.7	67,518	0.020	1.51	
0.8	77,192	0.015	1.26	
0.9	86,439	0.009	1.26	
1.0	94,354	0.004	1.05	

ROADS network. Even though this could represent an interesting result in specific application scenarios with less stringent requirements in terms of computation time, we remark that the objective of our paper is to support ahead monitoring of a large-scale transportation network. This objective imposes a near real-time requirement on the execution time and, therefore, demands for larger speedup values with respect to Brandes. From the reported speedup values, it appears that such objective can be achieved with our approach when using a K-fraction parameter lower than 0.4. As reported in Table 2, the average error of W2C-Fast-BC with K = 0.4 is very low (0.043%) for the top-1000 nodes, *i.e.,* the nodes with the highest BC values. The error increases when K is smaller. However, it is still less than 1%.

The following sub-sections aim at generalizing these preliminary results related to the performance of our approach, by specifically focusing on two aspects: *i)* assessing the accuracy and efficiency of our solution on static, directed and weighted graphs; *ii)* identifying a minimum tolerable threshold in terms of both execution time and percentage error with dynamic weighted graphs in the context of our case study. Given the inability of the available BADIOS implementation of treating weighted graphs and the relatively lower speedup achieved with respect to our solution on unweighted graphs, the rest of the performance analysis only considers our custom Scala implementation of Brandes for weighted graphs as benchmark. Both Brandes and W2C-Fast-BC have been implemented using the map-reduce framework, which naturally supports parallelism. In particular, the following evaluations leverage two Spark workers, each configured to use 5 cores for parallel execution.

Spark was configured to work in the standalone cluster mode on two Intel Xeon E5 2640 2.4 GHz multi-core machines, each equipped with 56 virtual cores and 128 GB of DDR4 RAM.

#### Static, directed, weighted graphs

As a first static graph, we exploit the original directed Rhone-ROADS graph, where edges are both directed and weighted according to the lengths (in meters) of the road segments. Thus, the computation of shortest paths through the Dijkstra algorithm reduces the BC of the nodes traversed by longer paths between pairs of nodes. We remark that the road-length does not account for traffic dynamics.

**Fig 8:**
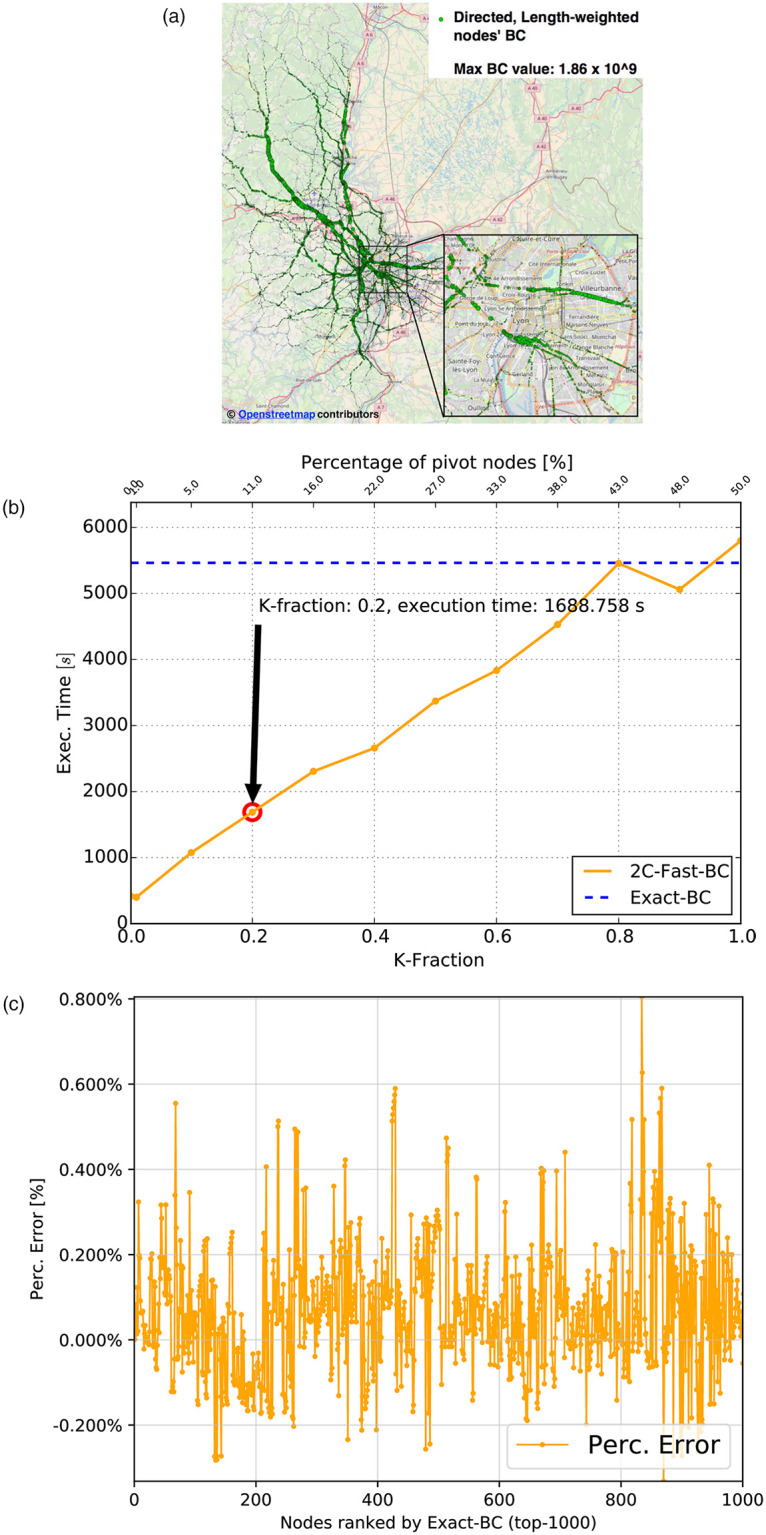
Directed graph weighted with discretized road-lengths. (a) Nodes’ BC values (circle size proportional to node’s BC) (b) Execution times (W2C-Fast-BC and Brandes BC) with 10 cores (c) Percentage error of W2C-Fast-BC with K-fraction = 0.2 (top-1000).

The values of nodes’ weighted BC via our novel W2C-Fast-BC algorithm is reported in Fig 8a. W2C-Fast-BC introduces an important speedup of approximately 3.2 (1,688 seconds against 5,465 seconds), with respect to the exact, weighted, Brandes-based computation of BC, when considering a K-Fraction = 0.2 (Fig 8b). Also, the percentage error (Fig 8c) remains under a |0.8%| threshold for the top-1000 BC values. The slightly lower speedup (3.2) obtained with K-Fraction = 0.2 on the road-length weighted graph with respect to the unweighted case (4.54, as from Table 2) can be explained by considering the higher complexity of the Dijkstra algorithm and the higher number of classes due to higher-variance of the shortest-path length. Finally, it is worth to highlight the crucial role that the second level of clustering plays in lowering the computation time by reducing the number of SSSP explorations. This can be appreciated by analyzing the number of pivots reported, as percentages of the total number of graph nodes, in the top *X*-axis of Fig 8b. In particular, when using a K-fraction = 1, the reduction in the number of SSSP explorations is only due to the class detection mechanism based on Louvain clustering for the retrieval of border nodes. In such case, 58,797 pivots *i.e.,* a fraction of approximately 50% of the graph nodes (117,605), is used to perform SSSP explorations, resulting in a total computation time larger than the one obtained with the Brandes algorithm, due to the overheads introduced by Louvain clustering and local BC computation that overcomes the benefit of a reduced number of SSSP explorations. However, by relying on the second level of clustering, even with rather high values of the K-fraction parameter (*i.e.,* 0.7—0.9), the number of pivots is reduced to 20-30% of the total number of graph nodes, thus permitting to achieve an improvement with respect to Brandes in terms of computation time. By further reducing the K-fraction parameter to 0.2, the number of pivots lowers to 12,795, *i.e.,* 11% of the total number of nodes, thus achieving the mentioned speed-up of 3.2 and achieving our requirements of near real-time computation.

**Fig 9:**
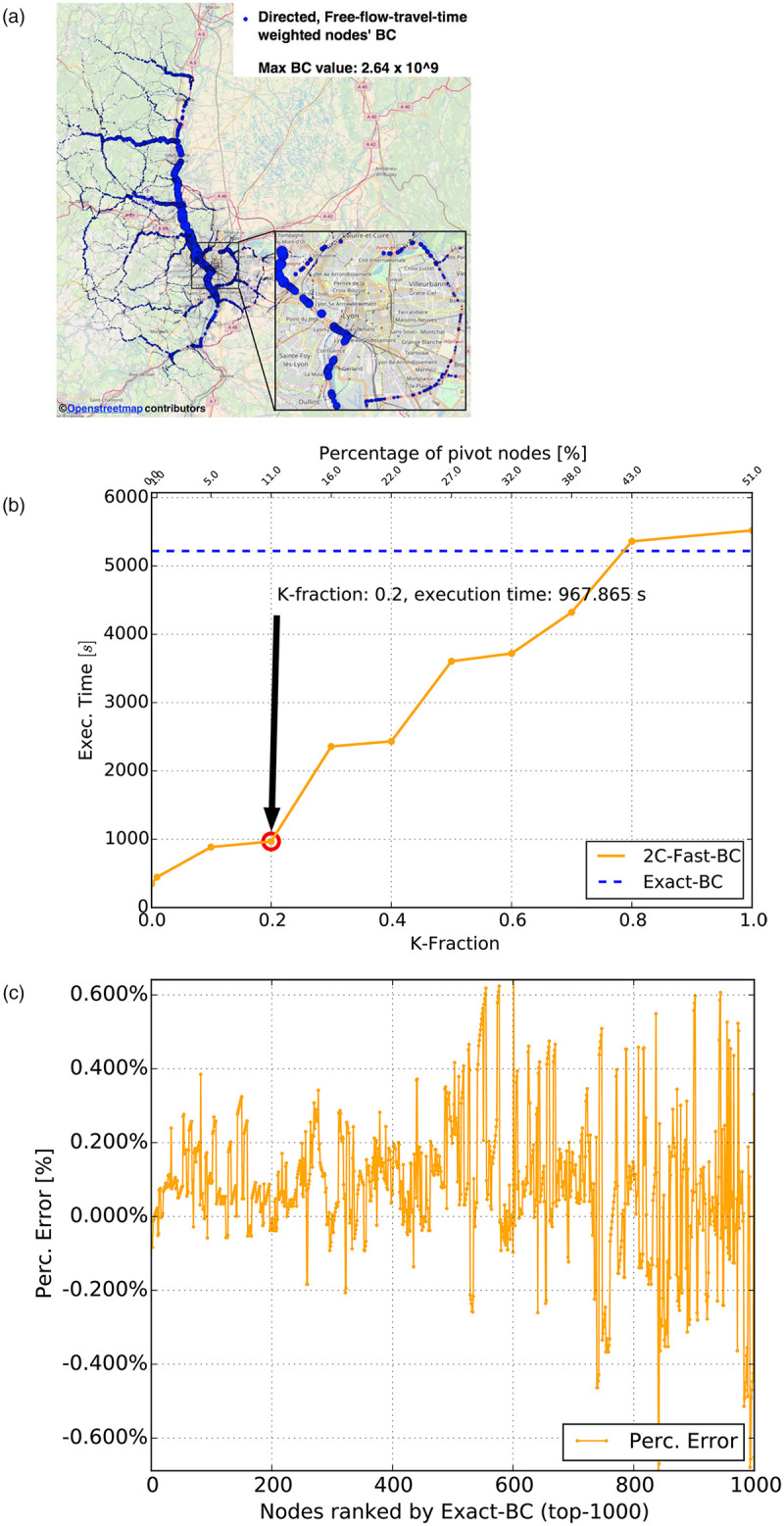
Directed graph weighted with discretized free-flow-travel-times. (a) Nodes’ BC values (circle size proportional to node’s BC) (b) Execution times (W2C-Fast-BC and Brandes BC) with 10 cores (c) Percentage error of W2C-Fast-BC with K-fraction = 0.2 (top-1000).

As a second static graph, we weigh edges by considering free-flow-travel-time (FFTT), an information easily derivable for all edges of the Rhone-ROADS network by dividing the road length by the road segment speed limit. FFTT-weighted BC values computed via the W2C-Fast-BC algorithm are reported in Fig 9a, with K-Fraction parameter equal to 0.2. It is worth noting that the analysis is related to free-flow traffic conditions, and therefore does not take into account transient situations (*e.g.,* accidents or congestions) that can deeply change the weight (*i.e.,* travel time) distribution on the edges.

The considerations on performance of road-length-weighted BC apply also to the FFTT-weighted BC, with a slightly higher speedup approximately equal to 5.2 and a |0.6%| bounded percentage error on the BC value for the top-1000 nodes. The number of SSSP explorations in this case corresponds to 12,727, *i.e.,* a 10.8% fraction of the total number of nodes.

#### Dynamic graphs

**Fig 10:**
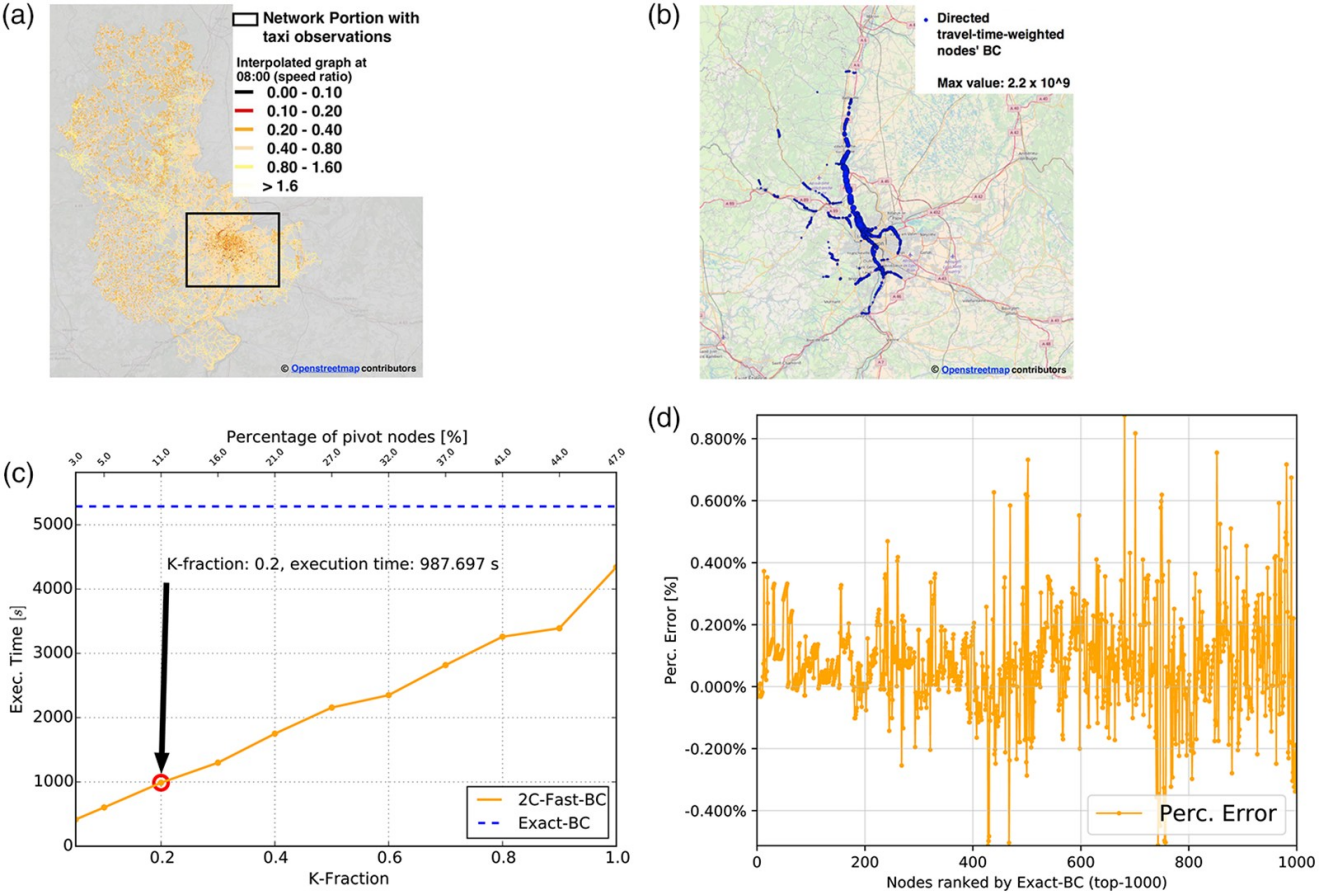
The interpolated dynamic taxi graph: Median-speed-to-max-speed ratio at different hours of the day. (a) KNR-interpolated graph at 08:00 (b) Top-1000 nodes’ BC values at 08:00 (c) Execution time of W2C-Fast-BC vs Brandes-BC at 08:00 (d) KNR-interpolated top-1000 BC percentage error at 08:00.

To obtain a dynamic, weighted network, larger than the one observed from taxi trips (*i.e.,* Rhone-OBS), we consider an interpolation technique aimed at estimating the hourly value of median speed (and therefore of travel-time) for those edges of the original Rhone-ROADS network with no available observation from taxi trips at time slot *t*. To that purpose, we use a non-parametric supervised machine-learning technique, namely *KNR-interpolation*, based on K-nearest-neighbor regression [60]. Each edge has been modeled as a data point with associated multiple features (*i.e.,* the attributes of Table 1 of the Rhone-ROADS network). The median speed, available for some edges (labeled instances) and missing for other ones (unlabeled instances) at a given time slot *t*, represents the feature we want to predict. We trained a K-nearest neighbors regressor on the labeled instances using the weighted Euclidean distance and the average criterion to predict the median speed for the unlabeled instances. In the weighted Euclidean distance, more distant data points are weighted less in the final regression formula to increase the robustness of the estimation. The value of K has been determined automatically via 10-fold cross-validation, typically resulting in the order of 40-50 neighbors. In Fig 10a, we graphically show the KNR-interpolated dynamic graph, in the snapshot related to 08:00. We remark that the interpolation has been performed over the whole typical day to construct the final dynamic graph. Fig 10a also presents, via the same color code used in Fig 4, the speed-ratios either estimated via taxi traces (for the framed portion of the graph) or via the KNR interpolation technique. The resulting graph has the same size of the Rhone-ROADS network and is therefore large enough to perform a relevant performance analysis, as the one presented in the static topological evaluation.

The top-1000 values of BC associated with this graph are reported in Fig 10b. As Fig 10c shows, the exact algorithm for computing BC on the weighted graph requires a computation time of more than one hour, therefore unable to complete within the duration of the time slot, thus making the computation of BC values at time slot 08:00 completely useless to provide any knowledge (*i.e.,* prediction of traffic flows for the next time slot) that could be exploited to inform travelers about congested roads. In comparison, our W2C-Fast-BC computes BC values in 987 seconds (*i.e.,* approximately 15 minutes) with a tolerable percentage error of 0.8% over the top-1000 BC nodes. The number of SSSP explorations performed on the 08:00 snapshot weighted graph corresponds to 12,727, *i.e.,* a 10.6% fraction of the total number of nodes. Similar results have been observed over the whole dynamic graph, thus proving the adequacy of our solution for quasi real-time monitoring of dynamic, weighted road-networks. The information derived from BC computation along with the related possible reorganization of traffic flows allow urban planners and managers to perform what we call as ahead monitoring, since it is possible to estimate where traffic is likely to be more intense in the near future.

#### Threats to validity

The W2C-Fast-BC solution proposed in this paper is subject to different sources of randomness, noise and measurement inaccuracies. In this section, we provide a discussion of the most important aspects that could impact the validity of BC-based estimation and the way such threats to validity have been addressed in this paper or will be investigated as matter of future work.

First of all, our solution is impacted by the randomness of the Louvain method and that of the K-means clustering, which are used, respectively: *i)* for the first level of clustering of our W2C-Fast-BC algorithm to identify border nodes and determine pivot nodes; *ii)* for the second clustering step aimed at grouping pivot nodes with similar properties with respect to the border nodes of a given cluster. We remind here that a low-quality first-level clustering could generate a higher number of border nodes thus increasing the chances of having external shortest paths, *i.e.,* shortest paths between nodes of a same cluster that include nodes outside the given cluster, a source of error in the computation of BC values with W2C-Fast-BC. To handle this aspect, our solution performs, in parallel, multiple runs of the Louvain method and selects the cluster set that corresponds to the highest value of the modularity index computed as part of the Louvain method. In our experiments, we set the number of concurrent iterations equal to 10, but this value can be further increased depending on the concurrency level allowed by the execution environment. This assures that the produced clusters are internally tight as well as loosely connected among one another. Selecting the configuration with higher modularity allows for reducing the number of border nodes. Concerning the second level of clustering, it is important to assure that the pivot nodes clustered together via K-means do present similar properties in terms of distance and number of shortest paths to border nodes to keep the error low when reducing the number of SSSP explorations. In this regard, we have considered a 0-tolerance threshold and a maximum number of allowed iterations in the order of hundreds of millions as the convergence criteria of the K-means algorithm in order to assure the best quality during this second level of clustering. As matter of future work, we are currently investigating more advanced solutions to: *i)* improve the quality of the first level of clustering, by exploiting algorithms that minimize the total edge cut between the different generated clusters, thus further reducing the number of border nodes. Solutions such as the well-known METIS algorithm [61] and its more recent parallel improvements [62] appears as a valid lead to pursue in order to achieve the aforementioned objective; *ii)* remove the error associated to classes of equivalence and the pivot mechanism [59].

**Fig 11:**
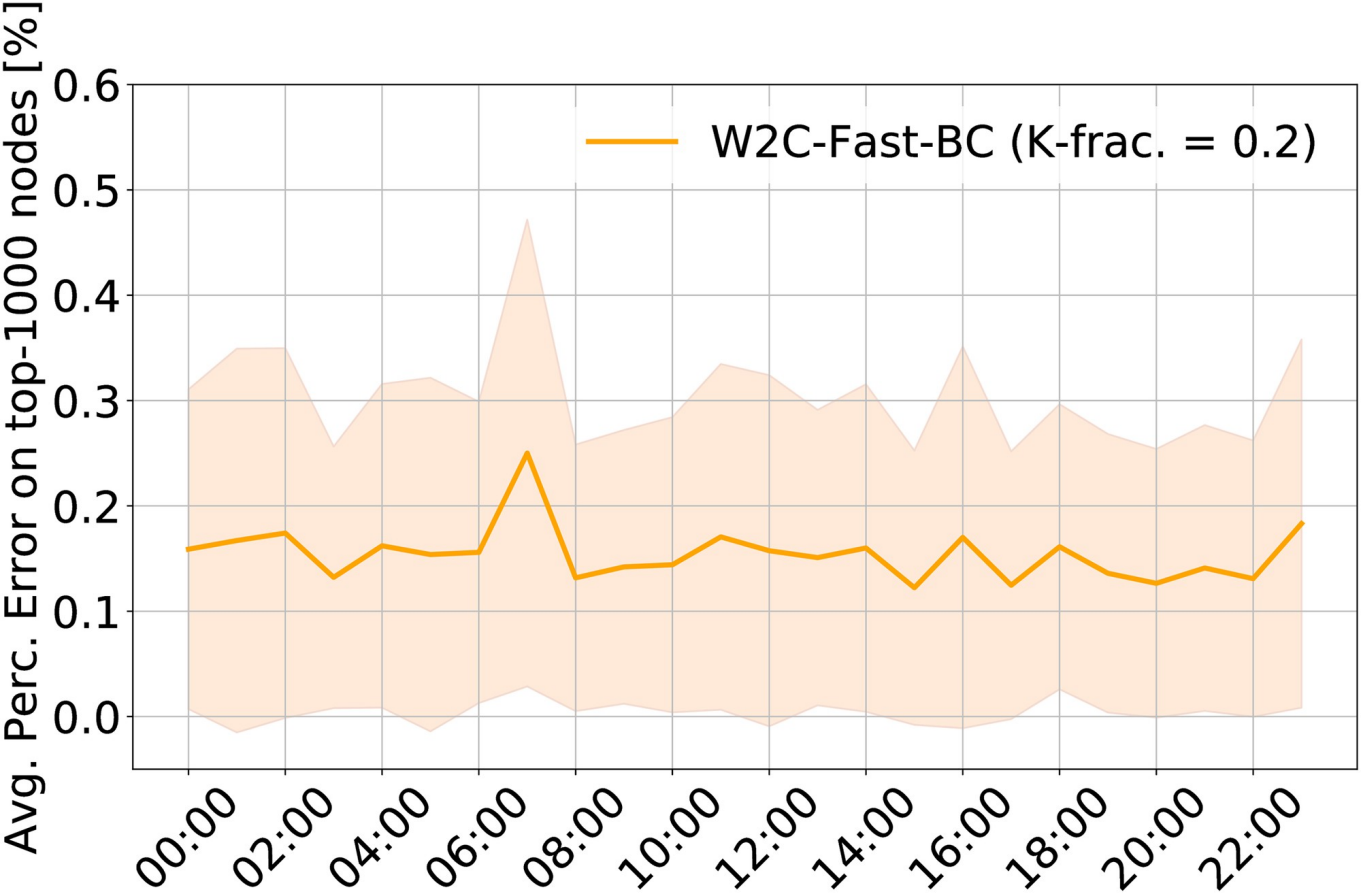
Dynamic evolution of the mean absolute percentage error with W2C-Fast-BC (K-fraction = 0.2) on the interpolated dynamic graph (on top-1000 nodes’ BC values). The shaded portion of the graph corresponds to measured values of standard deviation at each time step.

Concerning data availability, in our empirical evaluation we use consecutive GPS position observations from taxis on-board GPS sensors and the derived instantaneous speed measurements, which have been post-processed and associated to specific road segments of the underlying network via a map-matching solution. It is important to remind that we derive hourly typical travel-time graph weights by exploiting road length information and multiple speed observations related to a multitude of vehicles traversing each road segments, collected over different days. Nonetheless, samples can be unavailable or very limited due to low traffic flow, thus hampering the quality of the aggregate travel-time information and, therefore, the possibility of retrieving realistic hourly snapshots of the road network traffic dynamics, especially during non-peak hours and nighttime periods. Therefore, it appears realistic to study the accuracy of our W2C-Fast-BC solution over time and evaluate its robustness with respect to a varying number of available observations, as presented in Fig 2a and discussed in Section *BC on Dynamic Graphs*. In the following, we analyze the robustness of our solution W2C-Fast-BC for BC-estimation (with a K-fraction parameter set to 0.2) with respect to Brandes, when using a KNR-interpolated graph. The results are reported in Fig 11. With a mean absolute percentage error on the top-1000 highest-BC nodes bounded between 0 and 1%, the figure underlines the substantial insensitivity of the error to time variations and the related variability in the number of available data observations (see Fig 2a). Obviously, this is an effect largely due to the KNR-interpolation technique that allows identifying links with similar properties and retrieve values of the median speed variable for those one with missing observations.

Regarding noise, sensors are naturally subject to faults and inaccuracies that might generate imprecise, biased or anomalous measurements. In our context, position and speed measurements are acquired via civil-use GPS navigation systems, which are notoriously subject to several sources of error, largely studied in many papers from the related literature [63]. In order to account for the presence of noise in our empirical evaluation, we evaluate in the following the robustness of our W2C-Fast-BC solution with respect to Brandes when a simple model of the noise that can be present in the measurements acquired via GPS sensors is considered. To this purpose, we analyze stochastic variations of the previously described hourly weighted KNR-interpolated snapshot graphs, by considering the presence of an additive gaussian noise on the available travel-time measurements. More specifically, for a given KNR-interpolated hourly snapshot graph *G*_*t*_, we modify the travel-time weight *w*_*l*_, associated to the generic network link *l* of the graph *G*_*t*_, by drawing a random sample from a 0-centered Gaussian distribution $N_{l}$. In our experiments, we consider a different Gaussian distribution for each road link *l*, by setting its standard deviation to a fixed fraction Ψ of the link free flow travel time *fft_l_*, *i.e.,* $N_{l}\left(0,\Psi \cdot {\text{fft}}_{l}\right)$. By this choice, we therefore assume errors in GPS measurements generate an additive noise on the median travel time of each link from *G*_*t*_ and that such error depends on the free flow travel time of the road segment, *i.e.,* larger errors are more likely to take place on longer-to-travel road segments. The randomly sampled noise is thus added to *w*_*l*_, by capping possibly negative weights to the minimum observed travel time from the edge weights of *G*_*t*_. By repeating this operation for all the links of *G*_*t*_, we finally obtain a noisy instance ${\hat{G}}_{t}$ of the original snapshot graph that we use to estimate BC values via both W2C-Fast-BC and Brandes.

In Table 3, we report statistics related to the accuracy of W2C-Fast-BC with respect to Brandes, when both algorithms are leveraged to compute the BC values for all nodes of the noisy instances ${\hat{G}}_{t}$. Given the previous considerations on the insensitivity of the KNR-interpolated graph to the different availability of samples over the hours of the day, we focused our analysis on the KNR snapshot graph related to 08:00, *i.e.,* *G*_08:00_, to compute the noisy instances considered in our evaluation. The following statistical indicators are considered to evaluate the accuracy of BC estimation in the different noise scenarios: *i)* mean absolute percentage error for the top-1000 BC nodes, *ii)* number of BC nodes present in the exact top-1000 BC ranking, *iii)* normalized inversion count for the top-1000 BC nodes ranking. The inversion count of an array corresponds to the number of inversions required to sort the array. When considering the ranking obtained via an approximate BC computation algorithm, it represents the number of inversions required to correctly rank the array according to an exact BC computation. In the normalized version of the index, the count is divided by the maximum number of inversions, *i.e.,* $\frac{N*(N-1)}{2}$, for an array of size *N*. To perform a statistically relevant sensitivity analysis, we consider different values of the Ψ fraction (*i.e.,* 2.5, 5, 10 and 20), and 10 different random instances of ${\hat{G}}_{t}$ for each choice of Ψ. Results are averaged over the different realizations. The choice of the specified range for the Ψ fraction is motivated by the magnitude of the travel-time link error that derives from it. For instance, with a Ψ fraction equal to the smallest value of 2.5, we observe a variation of the travel time weights in the order of ±(5 to 10) seconds for approximately hundreds of links out of 248,337, on average. With a Ψ fraction equal to the largest value of 20, we observe instead a variation of the travel time weights in the order of ±(5 to 100) seconds for approximately 25,000 links out of 248,337, on average. The variations induced by the selected range of values for the Ψ parameter appear to reasonably cover a wide range of scenarios in terms of noise intensity. The statistics in Table 3 indicate that the accuracy of W2C-Fast-BC on the top-1000 nodes is not sensitive to the underlying variations of the graph when compared to Brandes, as the mean absolute percentage error remains extremely low and the ranking accuracy is very high (number of retained nodes and normalized inversion count) in all configurations. Additionally, such performance figure does not significantly variate with respect to the level of introduced noise. This is indeed an expected results of our algorithm, *i.e.,* our algorithm is capable of identifying the most critical nodes of the network very accurately, even in presence of noise that can change the spatial distribution of the most critical nodes of the network.

**Table 3:** Accuracy of W2C-Fast-BC with respect to Brandes on the noisy graph. Mean and standard deviation are reported for each accuracy indicator as obtained by aggregating over the 10 instances of the noisy graph ${\hat{G}}_{08:00}$ for each value of Ψ.

Gaussian noise Ψ	Top-1000 mean abs. perc. err. [%]		# of retained Top-1000 nodes		Normalized inversion count [%]	
	mean	std	mean	std	mean	std
2.5	0.14	0.01	997.80	1.69	0.28	0.03
5	0.13	0.02	998.10	1.73	0.28	0.03
10	0.14	0.02	998.20	1.14	0.31	0.04
20	0.14	0.02	998.50	0.85	0.30	0.03

To further investigate this aspect, we apply again both W2C-Fast-BC and Brandes on the ten random instances of ${\hat{G}}_{08:00}$ and compare the results obtained in the two cases with respect to the exact values of BC and rankings computed on the original snapshot graph *G*_08:00_. To that purpose, all the indicators in Table 3 have been recalculated using the values of BC obtained via Brandes on graph *G*_08:00_ as reference values. We underline that, in this context, the absolute percentage error does not represent a real error, but rather the percentage absolute difference with respect to the baseline BC values computed on the graph without noise. Similarly, the errors related to BC ranking only indicate that the most BC critical nodes are different in presence of noise (${\hat{G}}_{08:00}$) with respect to the base scenario (*G*_08:00_). Statistics for W2C-Fast-BC and Brandes are reported in Tables 4 and 5, respectively. The reported values clearly indicate that an increasing presence of noise (*i.e.,* higher Ψ fractions for the computation of the standard deviation of the normal distribution) determines a more relevant change in the distribution of BC values, thus significantly changing both the values of BC (as indicated by the percentage error) and the nodes that are most critical in terms of BC. In addition, the comparison of the statistics in the two tables highlights that our algorithm is only negligibly more sensitive to noise than Brandes, as the three reported indices are comparable up to the first decimal digit in almost all cases for the two algorithms.

**Table 4:** Sensitivity of W2C-Fast-BC to different levels of random noise introduced on *G*_08:00_.

Gaussian noise Ψ	Top-1000 mean abs. perc. err. [%]		# of retained Top-1000 nodes		Normalized inversion count [%]	
	mean	std	mean	std	mean	std
2.5	1.19	0.59	982.80	7.94	1.88	1.03
5	6.08	2.68	933.50	26.88	8.66	3.82
10	18.37	6.72	782.80	81.63	19.03	6.14
20	27.60	6.77	655.50	58.17	24.31	4.39

**Table 5:** Sensitivity of Brandes to different levels of random noise introduced on *G*_08:00_.

Gaussian noise Ψ	Top-1000 mean abs. perc. err. [%]		# of retained Top-1000 nodes		Normalized inversion count [%]	
	mean	std	mean	std	mean	std
2.5	1.15	0.60	983.00	8.14	1.80	1.02
5	6.07	2.68	933.60	27.29	8.58	3.83
10	18.35	6.71	782.60	81.91	18.96	6.14
20	27.57	6.79	655.50	58.71	24.23	4.39

The reported sensitivity analyses make possible to conclude that our W2C-Fast-BC solution with KNR-based interpolation is extremely accurate as well as robust to both limited availability of observations and presence of noise in the observations, when used to compute the BC of the most critical nodes of the network (*i.e.,* those with highest values of BC).

## Conclusion

In this paper, we have proven, through an in-depth analysis performed on a large real network, that betweenness centrality is a useful indicator of both structural bottlenecks (in static, unweighted and weighted graphs) and traffic conditions (in dynamic, weighted graphs). At the same time, we have pointed out that in a dynamic context the estimation of traffic flows requires fast computation of BC. A requirement that we satisfy with our algorithm able to compute good approximation of BC values in short times.

For the study, we have used two datasets: one related to the whole road network of Lyon, whose weights have been computed using free-flow travel times and road lengths, and another one containing GPS traces of taxi trips, with a partial coverage of the whole road network, for estimating dynamic weights from average travel times.

The results of BC computation over the network representing the Lyon metropolitan area (both in a static and dynamic scenario) confirm that our algorithm is very fast and at the same time able to keep the error below a desired threshold, so posing the basis for its exploitation as core component of a ahead monitoring system.

Possible improvements in terms of approximation errors will be evaluated in comparison with performance degradation due to the removal of the sources of error at the first clustering level with the integration of the new version of the algorithm aimed at computing the exact value of BC.

Our W2C-Fast-BC algorithm is planned to be plugged into a dynamic and adaptive distributed control system aimed at performing resilience enhancement by keeping, over space and time, the values of BC close to the ones observed in free-flow conditions so as to achieve a more uniform distribution of traffic flows and, consequently, guarantee more efficient network states at equilibrium.

In the future, we intend to extend our analysis by exploiting a dataset with a larger coverage for dynamic weights as, due to a limited coverage of GPS data, we have been forced to apply an interpolation technique based on non-parametric regression to realistically scale the dynamic analysis on a larger network. Moreover, we plan to evaluate and eventually integrate BC with other information related to a preventive or statistical knowledge of traffic distribution that could contribute to the definition of a more effective traffic predictor based on betweenness centrality.

## Supporting information

S1 File (TXT)

S1 Data (ZIP)

S2 Data (ZIP)

S3 Data (ZIP)

S4 Data (ZIP)

S5 Data (ZIP)

S6 Data (ZIP)

S7 Data (ZIP)
